# Alternate Phosphorylation/O-GlcNAc Modification on Human Insulin IRSs: A Road towards Impaired Insulin Signaling in Alzheimer and Diabetes

**DOI:** 10.1155/2014/324753

**Published:** 2014-12-17

**Authors:** Zainab Jahangir, Waqar Ahmad, Khadija Shabbiri

**Affiliations:** ^1^Department of Chemistry, GC University Lahore, Lahore, Pakistan; ^2^School of Biological Sciences, Goddard Building, The University of Queensland, Brisbane, QLD 4072, Australia

## Abstract

Impaired insulin signaling has been thought of as important step in both Alzheimer's disease (AD) and type 2 diabetes mellitus (T2DM). Posttranslational modifications (PTMs) regulate functions and interaction of insulin with insulin receptors substrates (IRSs) and activate insulin signaling downstream pathways via autophosphorylation on several tyrosine (TYR) residues on IRSs. Two important insulin receptor substrates 1 and 2 are widely expressed in human, and alternative phosphorylation on their serine (Ser) and threonine (Thr) residues has been known to block the Tyr phosphorylation of IRSs, thus inhibiting insulin signaling and promoting insulin resistance. Like phosphorylation, O-glycosylation modification is important PTM and inhibits phosphorylation on same or neighboring Ser/Thr residues, often called Yin Yang sites. Both IRS-1 and IRS-2 have been shown to be O-glycosylated; however exact sites are not determined yet. In this study, by using neuronal network based prediction methods, we found more than 50 Ser/Thr residues that have potential to be O-glycosylated and may act as possible sites as well. Moreover, alternative phosphorylation and O-glycosylation on IRS-1 Ser-312, 984, 1037, and 1101 may act as possible therapeutic targets to minimize the risk of AD and T2DM.

## 1. Introduction

Alzheimer's disease (AD) is a neurodegenerative disease associated with a progressive loss in memory and other mental processes, resulting in dementia. In the AD brain, some changes occur in tertiary structures of microtubule-associated protein tau and *β*-amyloid (A*β*) peptide resulting in self-association and deposition. Hyperphosphorylated forms of tau act as major constituents of neurofibrillary tangles (NFTs), while A*β* is major constituent of plaques [1]. By 2050, there is expected to be a million new cases of AD per year, or one new case every 33 seconds and AD prevalence is proposed to be 11 to 16 million [2]. On the other hand diabetes mellitus (DM) is characterized by hyperglycemia. Type 1 diabetes is insulin-dependent diabetes mellitus caused by absolute deficiency of insulin secretion while type 2 diabetes (T2DM) is non-insulin-dependent diabetes mellitus caused by both inadequate insulin secretion and insulin resistance of target organs [3]. In DM, numerous peripheral organs, such as kidneys, eyes, and peripheral nerves, undergo pathological changes. DM also affects the central nervous system (CNS). Specifically, both types of diabetes are associated with impairment of learning abilities and memory deficits [3]. Hoyer in 1998 presented a hypothesis stating that brain insulin may be resisted in AD and that AD can be called a brain specific “type 3 diabetes.” Both AD and T2DM are degenerative diseases characterized by neuronal loss and *β*-cells destruction, respectively. Impaired insulin signaling resulting in neurodegeneration and cognitive damages is the main systematic link between these two diseases. Many studies found that DM almost doubles the risk of AD. It was also found that diabetes is not only the risk factor of AD but it also accelerates the onset of AD. Recent studies also elaborate the involvement of impaired insulin signaling in tau hyperphosphorylation. AD and DM also share several enzymes (DOPA decarboxylase, glutamic acid decarboxylase) and growth factor receptors (thyrotrophin-releasing hormone, neuronal growth factor receptors, and p75 receptors) [4–9].

Insulin receptor substrate proteins (IRSs) act as interaction platforms that are phosphorylated on multiple tyrosine residues and attract different signaling proteins to spread the stimulus. Two isoforms of IRSs, that is, IRS-1 and IRS-2, are most abundant in mammals and have many tyrosine phosphorylation sites. In general, IRS-1 is important for growth, while proper function of pancreatic *β*-cells and glucose homeostasis are main functions of IRS-2 [10, 11]. Knockout of either of them causes insulin resistance, whereas only IRS-2-knockout results in diabetes. Either IRS-1 or IRS-2 gene deletion in mice leads to insulin resistance [12]. Irreconcilably, heterozygous and brain specific deletion of IRS-1 and IRS-2 in mice increases duration of life [13, 14]. However, IRS-2 deletion is important to induce tau phosphorylation and it also accumulates cytoplasmic deposits of phosphorylated tau [15]. Loss of IRS-2 in neurons of AD patients is particularly severe. IRS-1 and IRS-2 decreased levels in AD neurons increase NFT pathology. Reduced IRS-1 and IRS-2 levels indicate ineffective signaling of IR and IGF-1R in AD [16]. Apart from their important role in metabolic signaling, IRSs also spread proliferative and antiapoptotic signals and as a result become overexpressed in most cancers [17].

In biological systems, posttranslational modifications (PTMs) determine protein activity, localization, and their interaction with other proteins [18]. Multiple kinases activated by many triggers of insulin resistance, like reactive oxygen species [19], inflammatory cytokines [20], or excess lipids [19, 20], phosphorylate IRSs on several Ser/Thr residues [20]. This phosphorylation on various Ser/Thr residues is thought to inhibit insulin signaling and induce insulin resistance by blocking IRSs Tyr phosphorylation. Increased levels of inactivated phosphoserine-312-IRS-1 and phosphoserine-616-IRS-1 associated with decreased levels of IRS-1 and IRS-2 were identified in AD neurons. These increased levels of inactivated phosphoserine are strongly colocalized with NFTs [16]. Phosphorylation of Ser or Thr residue results in IRS-1 or IRS-2 inactivation [21]. IRS-1 after Ser/Thr phosphorylation becomes a weak substrate for enzyme insulin receptor tyrosine kinase [22–24]. Phosphoserine-312-IRS-1 causes IRS-1 inactivation by disordering IRS-1 binding to insulin receptor (IR). However, phosphoserine-616-IRS-1 prevents insulin activation of PI3-kinase [21, 23, 25]. Mechanistically many kinases that are overexpressed in AD phosphorylate IRS-1 result in IRS-1 inactivation and IR/IGF-1R resistance. Phosphorylation and O-glycosylation are also involved in affinity changes of IGFBP-6 and cause inhibition of IGF-II action on target cells [26, 27]. O-glycosylation of IGFBP-6 leads to 10-fold higher affinity for IGF-II by preventing IGFBP-6 binding to glycosaminoglycans and cell membrane [28].

Analogous to phosphorylation, O-glycosylation is also one of the important PTMs during which N-acetyl-glucosamine (O-GlcNAc) molecule is introduced on Thr or Ser residue by O-GlcNAc transferase (OGT). Phosphorylation on Thr or Ser residue can be inhibited by the addition of O-*β*-GlcNAc. Interplay between these two PTMs on the same residues also known as yin yang sites has been reported in several nuclear and cytoplasmic proteins [29]. These PTMs regulate many functions of the proteins and are responsible for temporary conformational changes [30]. Various studies have shown that O-glycosylation on IRSs reduced in AD and T2DM due to impaired insulin signaling and glucose metabolism [31–34]. Although some Ser/Thr sites are mapped to be O-glycosylation on C-terminal of IRSs, their exact location is still undetermined. This work predicts potential phosphorylation, O-*β*-GlcNAc, and yin yang sites on IRS-1 and IRS-2 proteins, involved in metabolic functions in the brain by using various bioinformatics tools. Impaired phosphorylation and O-glycosylation on IRS 1 and 2 lead to defective insulin signaling that resulted in Alzheimer- and diabetes-like complications. The sites where this type of crosstalk occurs are useful targets for designing new drugs for resulting diseases.

## 2. Materials and Methods

### 2.1. Multiple Alignment and Sequences Retrieval

The FASTA sequences of human IRS-1 and IRS-2 were taken from the Uniprot sequence database (http://www.uniprot.org/uniprot/?query=irs&sort=score) with entry name IRS1_HUMAN and accession number P35568 and entry name IRS2_HUMAN with accession number Q9Y4H2, respectively. Blast of these two sequences was made by Blast/Uniprot. A total of 37 hits for IRS-1 and 24 hits for IRS-2 were obtained with* E*-value of zero or less. Sequences based on uncharacterized, putative, hypothetical, predicted, and unnamed proteins were neglected. For the multiple alignments of IRS-1 proteins, eight sequences were selected from 37 retrieved sequences. The accession numbers of these selected sequences are given in Table 1. For the alignment of IRS-2 proteins, three sequences were selected from 24 retrieved sequences. The accession numbers of these three selected sequences are given in Table 2. ClustalW (http://www.ebi.ac.uk/Tools/msa/clustalw2/) was used for the multiple alignments of all the sequences of IRS-1 and IRS-2 to get the conservation status of serine, threonine, and tyrosine residues.

### 2.2. Protein Structure Prediction and Analysis

As there were no complete 3D structures of human IRS 1 and IRS 2 available in protein data bank, ab initio models were designed by using software MODELLER (ModWeb: https://modbase.compbio.ucsf.edu/scgi/modweb.cgi) [35], I-TASSER (http://zhanglab.ccmb.med.umich.edu/I-TASSER/) [36], and Swiss Model (http://swissmodel.expasy.org/) [37].

On the basis of Ramachandran plots, best ab initio models were selected. Ramachandran plots were taken from MolProbity (http://molprobity.biochem.duke.edu/) server [38]. FATCAT web server (http://fatcat.burnham.org/fatcat-cgi/cgi/fatcat.pl?-func=pairwise) was used to perform pairwise alignment of protein 3D structure. Chimera (http://www.cgl.ucsf.edu/chimera/) [39] and Jmol (http://jmol.sourceforge.net/) software were used to view and analyze 3D structure.

### 2.3. Prediction Methods for PTMs

We used three distinct algorithms: support vector machine (SVM), neural network (ANNs), and hierarchal searches [40] to reduce the false negative and false positive sites during predicting PTM sites on human IRS-1 and IRS-2. The NetPhos server generates neural network predictions for Ser/Thr/Tyr phosphorylation sites in eukaryotic proteins. The NetPhosK 1.0 produces kinase specific phosphorylation sites predictions. KinasePhos is a new version of kinase-specific phosphorylation site prediction server. Phospho.ELM is a database of experimentally confirmed phosphorylation sites in eukaryotic proteins. The YinOYang server predicts O-*β*-GlcNAc attachment sites in eukaryotic nuclear and intracellular protein sequences. These bioinformatics tools have been used efficiently in many studies [27, 41, 42].

#### 2.3.1. Phosphorylation Residues and Related Kinases Prediction

Neural network-based programs NetPhos 2.0 (http://www.cbs.dtu.dk/services/NetPhos/) [43] and Scansite (http://scansite.mit.edu/motifscan_id.phtml) [44] were used to predict phosphorylation potential for human IRS 1 and IRS 2 for serine, threonine, and tyrosine residues. 0.5 is minimum threshold value for NetPhos 2.0 used to predict phosphorylation.

Prediction of kinase specific phosphorylation sites in human IRS 1 and IRS 2 was made by Scansite (http://scansite.mit.edu/motifscan_id.phtml) [44], NetPhosK 1.0 (http://cbs.dtu.dk/services/NetPhosK/) [45], and KinasePhos 2.0 (http://kinasephos2.mbc.nctu.edu.tw/) [46]. Kinase specific acceptor substrates including Thr, Ser, and Tyr are predicted by these programs.

Phospho.ELM (http://phospho.elm.eu.org) [47] and Uniprot (http://www.uniprot.org/uniprot/) databases were used to obtain the data regarding experimentally verified phosphorylation sites on human IRS 1 and IRS 2.

#### 2.3.2. O-Glycosylated Residues and Yin Yang Sites Prediction

YinOYang 1.2 (http://www.cbs.dtu.dk/services/YinOYang/) database [48] was used to predict* O-β*-GlcNAc modification potential sites. The potential phosphorylation sites can also be predicted by YinOYang 1.2 program. Hence, this program predicts the yin yang sites with highly uneven threshold that is adjusted in accordance with amino acid surface accessibility. This method also helps to predict the potential yin yang sites. Modification potential and conservation status of Thr and Ser residues were used to determine the false negative (FN) yin yang sites.

The surface accessibility of predicted Ser, Thr, and Tyr residues for posttranslational modifications was assessed by using NetSurfP (http://www.cbs.dtu.dk/services/NetSurfP/) [49]. Scansite (http://scansite.mit.edu/motifscan_id.phtml) [50] was also used to check surface accessibility of human IRS 1 and IRS 2 to solvents and for PTMs.

## 3. Results

### 3.1. Alignment of Sequences to Determine the Conservation of Ser/Thr/Tyr Residues within IRS-1

**Figure 1:**
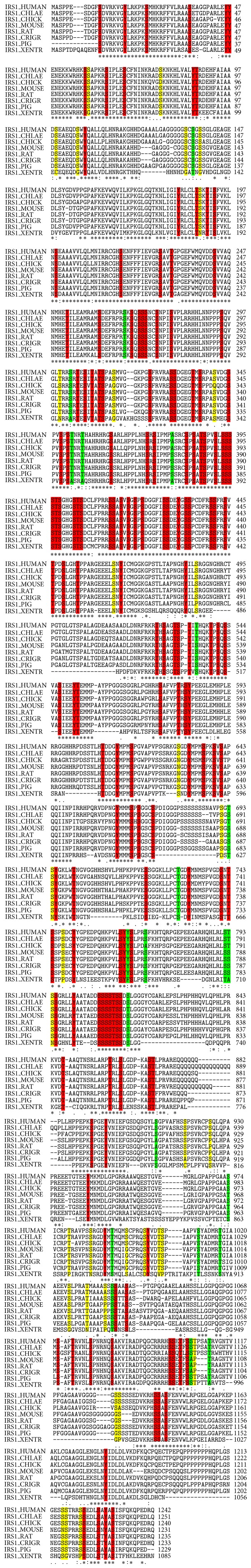
Multiple alignments of eight sequences (human, green monkey, pig, chicken, Chinese hamster, mouse, rat, and western clawed frog). ClustalW was used to align these different sequences. The conserved sequence (highlighted red) is marked by an asterisk, semiconserved (highlighted yellow) substitution by a single dot, and conserved substitution (highlighted green) by a double dot. Multiple alignment showed that Ser-11, 24, 36, 57, 63, 68, 193, 199, 217, 228, 243, 261, 270, 272, 273, 274, 277, 281, 295, 307, 312, 323, 329, 330, 337, 345, 348, 350, 362, 372, 374, 383, 385, 388, 391, 393, 394, 395, 396, 398, 402, 404, 412, 413, 415, 417, 419, 421, 427, 428, 433, 434, 440, 441, 444, 449, 527, 531, 541, 543, 547, 574, 581, 616, 636, 639, 641, 666, 668, 672, 720, 741, 744, 763, 766, 795, 807, 808, 809, 810, 812, 813, 862, 869, 892, 1038, 1041, 1070, 1078, 1084, 1100, 1101, 1105, 1142, 1143, 1145, 1223, 1227, and 1231; Thr-88, 188, 191, 231, 252, 305, 309, 311, 335, 387, 397, 403, 446, 453, 525, 530, 533, 539, 552, 579, 608, 700, 743, 811, 847, 859, 870, 1073, and 1103; and Tyr-18, 46, 47, 87, 107, 183, 431, 465, 483, 551, 582, 612, 632, 662, 695, 732, 750, 764, 800, 896, 941, 989, 1001, 1179, and 1229 are highly conserved residues. Meanwhile, Ser-137, 268, 303, 380, 770, 792, 815, 910, 974, 1011, 1037, and 1106; Thr-351, 354, 535, 693, 729, 793, 991, 1017, and 1111; and Tyr-765 showed conserved substitutions. Ser-58, 78, 99, 105, 135, 139, 189, 315, 341, 463, 486, 629, 691, 694, 747, 794, 918, 925, 985, 995, 1000, 1005, 1025, 1035, 1131, 1132, 1217, 1218, and 1222; and Thr-300, 979, 1004, 1030, and 1107 showed semiconserved substitutions.

To determine the evolutionary conservation of Ser/Thr/Tyr residues, IRS-1 was aligned with orthologous sequences of other species including green monkey, pig, chicken, Chinese hamster, mouse, rat, and western clawed frog (Figure 1). Out of 182 Ser residues, 99 (54.3%) were conserved, 12 (6.5%) were conserved substitute, and 29 (15.9%) were semiconserved. For total 60 Thr residues, 29 (48.3%) were conserved, 9 (15%) were conserved substitutes, and 5 (8.3%) were semiconserved. Same as for total 32 Tyr residues where 25 (78.1%) were conserved and 1 (3.1%) was conserved substitution. This data showed that human IRS-1 has high conservation status.

### 3.2. Alignment of Sequences to Determine the Conservation of Ser/Thr/Tyr Residues within IRS-2

**Figure 2:**
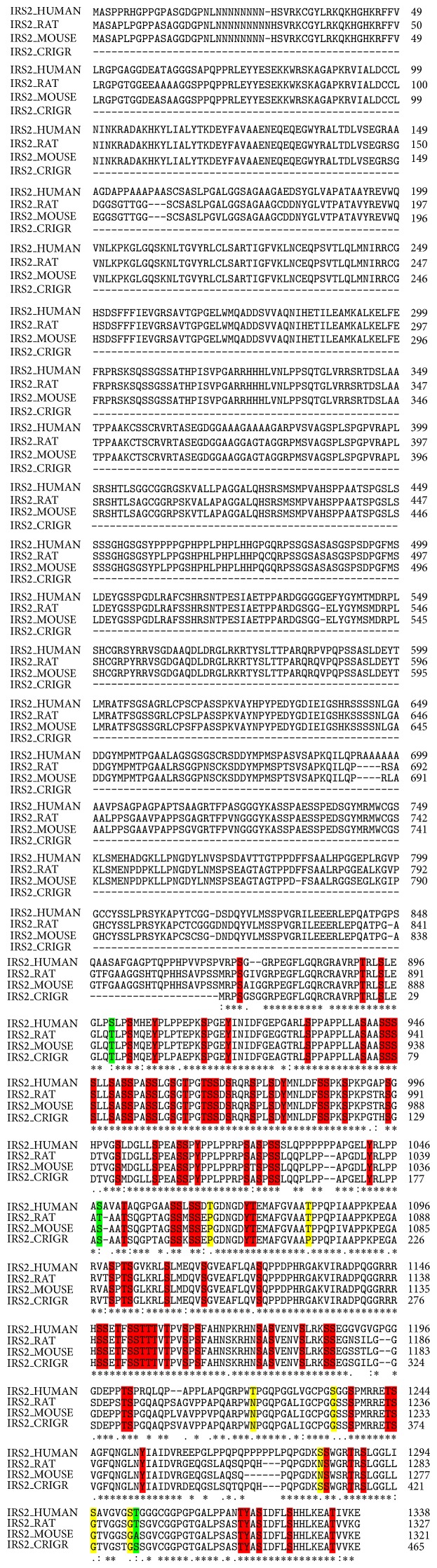
Multiple alignments of three sequences (human, rat, and mouse). ClustalW was used to align these different sequences. The conserved sequence (highlighted red) is marked by an asterisk, semiconserved (highlighted yellow) substitution by a single dot, and conserved substitution (highlighted green) by a double dot. Ser-3, 30, 66, 78, 84, 144, 162, 164, 166, 174, 210, 222, 237, 251, 253, 262, 277, 304, 306, 308, 309, 311, 312, 318, 334, 342, 346, 358, 365, 384, 388, 391, 400, 402, 406, 414, 428, 430, 432, 438, 444, 447, 449, 450, 451, 452, 456, 458, 482, 483, 485, 487, 489, 491, 493, 499, 505, 506, 515, 518, 523, 550, 560, 577, 591, 592, 594, 606, 608, 615, 619, 620, 639, 642, 643, 644, 645, 669, 672, 679, 682, 684,, 714, 730, 731, 735, 736, 740, 749, 752, 770, 772, 785, 804, 805, 809, 827, 828, 868, 873, 894, 903, 915, 932, 941, 944, 945, 946, 947, 950, 952, 953, 956, 957, 960, 966, 967, 969, 973, 976, 984, 985, 988, 995, 1001, 1007, 1011, 1012, 1022, 1024, 1026, 1027, 1060, 1061, 1063, 1064, 1100, 1103, 1109, 1115, 1124, 1148, 1149, 1153, 1154, 1162, 1164, 1174, 1176, 1181, 1185, 1186, 1203, 1237, 1244, 1283, 1289, 1322 and 1327; Thr-117, 140, 191, 214, 225, 239, 265, 286, 314, 336, 344, 350, 363, 404, 443, 520, 527, 575, 579, 580, 599, 604, 657, 719, 776, 779, 844, 859, 891, 962, 965, 1052, 1073, 1082, 1102, 1151, 1155, 1156, 1157, 1159, 1202, 1243, 1287, 1319, and 1334 and Tyr-36, 75, 76, 111, 116, 121, 136, 184, 194, 217, 459, 503, 540, 542, 556, 576, 598, 625, 628, 632, 653, 675, 727, 742, 766, 803, 810, 823, 907, 919, 978, 1014, 1042, 1072, 1253, and 1320 are highly conserved residues. Meanwhile, Ser-357, 848, 900, and 1048 and Thr-61, 544, 777, 815, and 1302 showed conserved substitutions. Ser-14, 183, 555, 665, 667, 704, 723, 820, 852, 1234, 1282, 1295, and 1301 and Thr-713, 1066, and 1221 showed semiconserved substitutions.

IRS-2 was aligned with only known orthologous sequences of species rat and mouse (Figure 2). Like IRS-1, human IRS-2 showed high conservation among different species. Out of 177 Ser residues, 159 (89.8%) were conserved, 4 (2.25%) were conserved substitutions, and 13 (7.34%) were semiconserved. While for total 53 Thr residues, 45 (84.9%) were conserved, 5 (9.4%) were conserved substitutes, and 3 (5.6%) were semiconserved. Tyr residues showed maximum conservation status. Out of 37 Tyr residues, 36 (97.29%) were conserved.

### 3.3. Predicted 3D Structures of Human IRS-1 and IRS-2 Proteins

3D structure of protein plays important role in determining the exact and precise functions of any protein. However, the whole 3D structures of IRS-1 and IRS-2 proteins have not been determined yet. To assess this difficulty we predict the 3D structure of these proteins by homology and ab initio modeling. We used many servers to predict the whole structure of both proteins and I-TASSER was the only server that predicts the complete structure of these proteins. I-TASSER predicted five models for each 3D structure with minimum *z*-score. The energy of these structures was minimized using YASARA software. To determine whether these structures truly represent or are nearer to possible future true structures, their geometry had been accessed by drawing Ramachandran plots that represent most reliable and suitable source to check predicted 3D structure's geometry. The Ramachandran plot analysis is a method to determine the phi and psi torsion angles between amino acids and compare this information to experimentally verified data as well as standard geometrical properties of protein 3D structures. Five models each were predicted by I-TASSER for constructing Ramachandran plots assessing both IRS-1 and IRS-2. Figures 7(a) and 7(b) represent the predicted 3D structures, Ramachandran plot analysis, and geometrical properties of IRS-1 and IRS-2.

**Figure 3:**
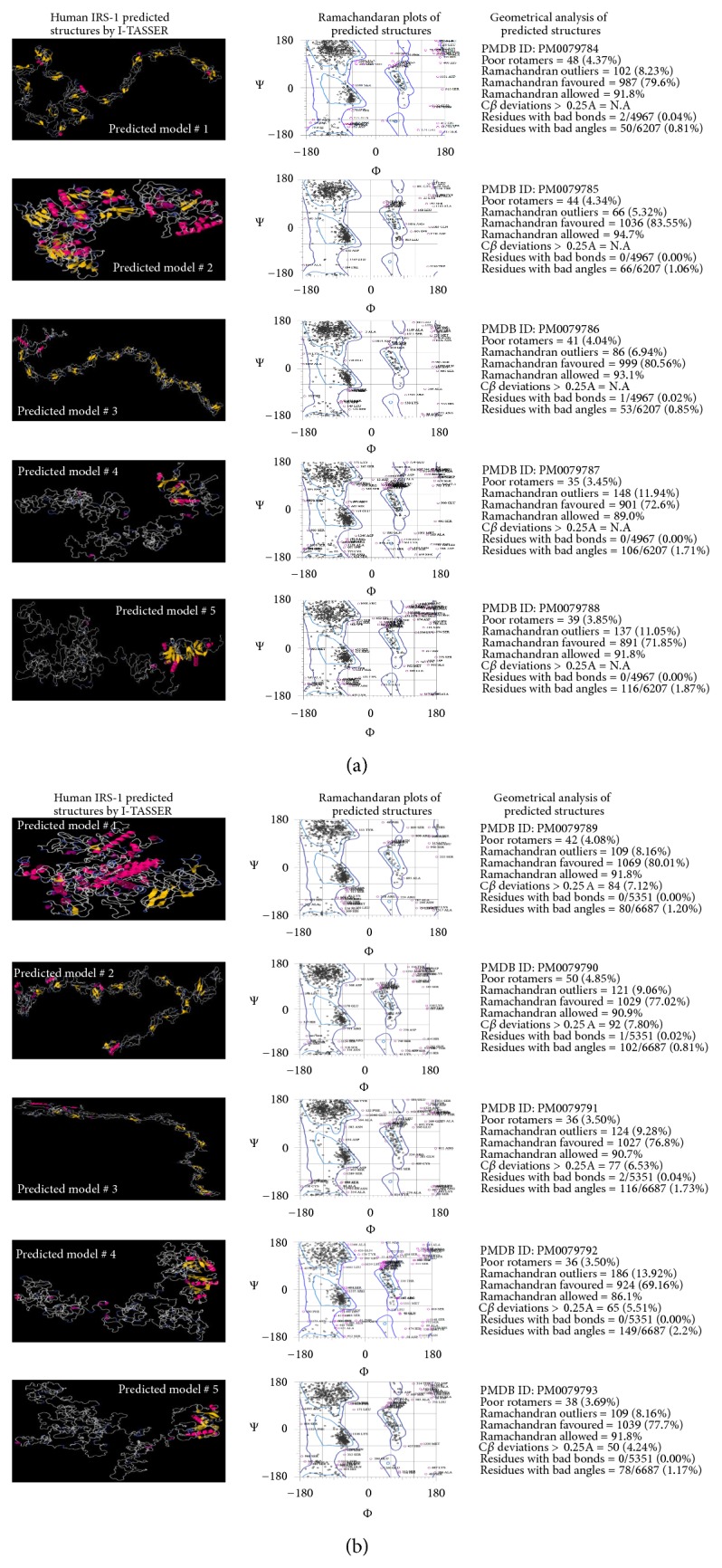
Homology models of human IRS-1 and IRS-2 retrieved through I-TASSER. Five models were predicted as possible 3D structures for both IRS-1 and IRS-2 proteins. The best model for each protein was selected on the basis of Ramachandran plots and their geometrical configuration. (a) Human IRS-1 full-length predicted 3D structures by I-TASSER with Ramachandran plots and geometry. (b) Human IRS-2 predicted 3D structures by I-TASSER with Ramachandran plots and geometry.

On the basis of Ramachandran plot analysis we proposed predicted model #2 (Figure 3(a)) as possible 3D structure for IRS-1 protein. In this structure 94.7% residues were in Ramachandran allowed region with only 5.32% outliers. No residues with bad bonds were found while only 1.06% residues with bad angles were predicted. However, the c*β* deviations for these predicted models were not predicted by server.

Ramachandran plot analysis was also performed for predicted structures for IRS-2 protein as shown in Figure 3(b) and model #1 was selected as best predicted model. The percentage of compliance for the predicted structure of IRS-2 with the Ramachandran plot indicates that 82.2% (361) of residues reside in the favored region while 94.3% (414) of all residues exist within the allowed region with only 9 C*β* deviations > 0.25A. Only 5.69% of residues exist within the disallowed region of the plot. The 3D structures of IRS-1 and IRS-2 proteins predicted by I-TASSER were submitted to online database PMDB—Protein Model Database (http://bioinformatics.cineca.it/PMDB/). PMDB accession numbers are given in front of each respective model in Figures 3(a) and 3(b).

**Figure 4:**
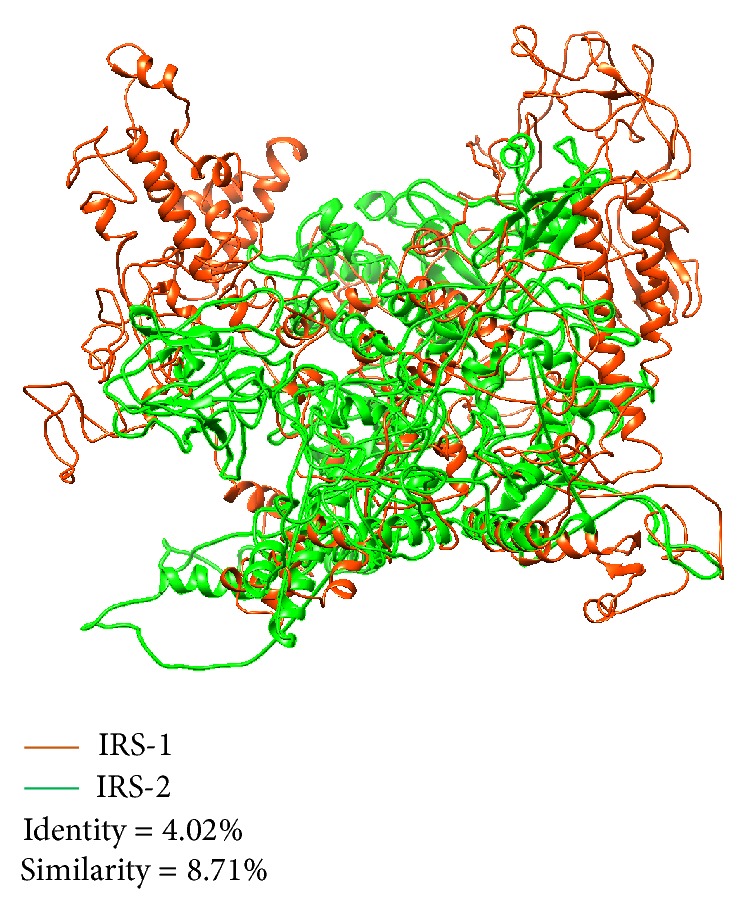
Homology models of both human IRS-1 and IRS-1 were retrieved utilizing automated protein modelling options through I-TASSER. The best 3D representative models for both IRS-1 (in red) and IRS-2 (in green) were aligned using FATCAT server and viewed in Chimera software package. Sequence alignment revealed that both proteins are significantly different in structure and only have 4.02% identity and 8.71% similarity.

IRS-1 and IRS-2 predicted 3D structures had been compared with each other by FATCAT and visualized by using Chimera as shown in Figure 4. IRS-1 structure was shown in red while IRS-2 in green. Both structures were significantly different (*P* value 9.01 E-01; Raw score = 251.71). The structure alignment has 546 equivalent positions with an RMSD of 11.03, with five twists. Both structures were found to have identity 4.02% and similarity 8.71%. This information leads to the conclusion that both IRS-1 and IRS-2 proteins regulate insulin singling in different ways.

### 3.4. Posttranslational Modification Prediction and Analysis

#### 3.4.1. Experimentally Verified Phosphorylation Sites on IRS-1 and IRS-2

Phospho.ELM is the website that contains data about experimentally verified phosphorylated residues and kinases involved in proteins to ease research work. Human IRS-1 has more than 40 Ser, Thr, and Tyr residues that were found to be phosphorylated experimentally. These include Ser-24, 268, 270, 272, 274, 307, 312, 323, 341, 345, 348, 374, 527, 531, 574, 616, 629, 636, 639, 794, 892, 1078, 1101, 1145, 1222, and 1223 Thr-530, and 1111, Tyr-46, 612, 662, and 896. Uniprot database also showed phosphorylation potential on Ser-3, 99, 329, 1223, and Tyr-465, 989, and 1229 on basis of similarity. Different kinases and groups of kinases like CK2, PLK1, IKK, PKC, MAPK3, SIK, AMPK, INSR, and RSK1 were involved in phosphorylation of IRS-1 on same or different residues.

For human IRS-2 protein phosphorylation had been verified on Ser-304, 306, 312, 334, 346, 365, 384, 388, 391, 518, 523, 550, 560, 577, 594, 608, 620, 679, 714, 730, 731, 735, 736, 770, 772, 828, 894, 915, 973, 1100, 1103, 1109, 1148, 1149, 1162, 1174, 1176, 1203, and 1283, Thr-350, 363, 520, 527, 713, 777, 779, 1151, 1159, and 1202, and Tyr-75, 184, 653, 675, 823, and 919 residues. While IKK kinase group was involved in phosphorylation, Uniprot database showed phosphorylation on four more residues including above, that is, Thr-527, 579, 580 and Tyr-1253.

#### 3.4.2. Predicted Phosphorylation Sites in IRS-1

**Figure 5:**
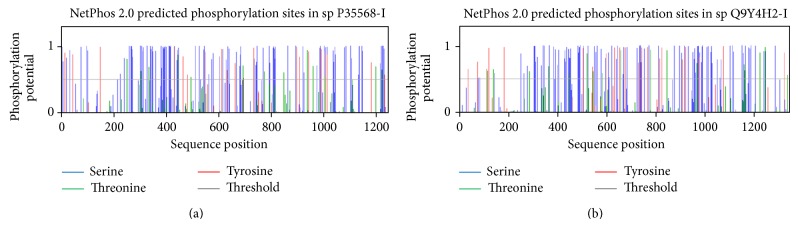
Graphical representation of the potential Ser, Thr, and Tyr residues for phosphorylation sites in human IRS-1 and IRS-2. (a) Predicted potential sites for phosphorylation on Ser, Thr, and Tyr residues. The light gray horizontal line shows the threshold for modification potential. The blue, green, and red vertical lines indicate the potential phosphorylated Ser, Thr, and Tyr residues, respectively. This graph was generated by NetPhos 2.0 web based program. (b) Predicted potential sites for phosphorylation on Ser, Thr, and Tyr residues. The light gray horizontal line shows the threshold for modification potential. The blue, green, and red vertical lines indicate the potential phosphorylated Ser, Thr, and Tyr residues, respectively. This graph was generated by NetPhos 2.0 web based program.

To predict phosphorylated residues in human IRS-1, NetPhos was used.  Figure 5(a) is a graphical representation of phosphorylation potential of the Ser, Thr, and Tyr residues in IRS-1. NetPhos predicted phosphorylation on 113 Ser, 13 Thr, and 20 Tyr residues including all experimentally verified phosphorylation sites except Ser-323, 374, 794 and Thr-530 and 1111 as shown in Table S1 available online at http://dx.doi.org/10.1155/2014/324753.

#### 3.4.3. Predicted Phosphorylation Sites in IRS-2

Similar to IRS-1, web server NetPhos 2.0 also made prediction of phosphorylated residues in human IRS-2.  Figure 5(b) is a graphical representation of phosphorylation potential of the Ser, Thr, and Tyr residues in IRS-2. These predicted phosphorylation residues include all experimentally confirmed sites except Ser-312, 388, 518, 679, 714, 722, 730, 772, 828, 1103, 1179, Thr-350, 527, 579, 713, 1151, 1202, and Tyr-75 as shown in Table S2.

#### 3.4.4. Predicted and Experimentally Verified Kinases in IRS-1

Various kinases involved in phosphorylation of human IRS-1. NetPhosK 1.0, Scansite, and KinasePhos 2.0 were used to assess possible kinases on IRS-1. The results obtained from these three servers had shown the involvement of various kinases in phosphorylation of human IRS-1. GSK3, PKC, and PKA showed maximum involvement in phosphorylation while other kinases like PKG, p38MAPK, cdk5, CKII, cdc2, ATM, and CKI also showed potential for phosphorylation of many Ser and Thr residues. INSR, syk, SRC, and insulin receptor kinase are the most predicted kinases for tyrosine phosphorylation as shown in Table 1S.

#### 3.4.5. Predicted and Experimentally Verified Kinases in IRS-2

Similar to IRS-1, NetPhosK 1.0, Scansite, and KinasePhos 2.0 predicted many kinases involved in phosphorylation of human IRS-2. GSK3, PKC, PKA, 14-3-3 Mode 1, PKG, p38MAPK, cdk5, CKII, cdc2, ATM, and CKI are the most predicted kinases involved in phosphorylation of many serine and threonine residues in IRS-2. INSR, syk, SRC, and insulin receptor kinase are the most predicted kinases for tyrosine phosphorylation as shown in Table 2S. Intrinsic tyrosine kinase in the *β*-subunit of IR is known to phosphorylate diverse substrates, including IRS-2 [51].

#### 3.4.6. Prediction of O-*β*-GlcNAc Modification and Potential Yin Yang Sites for IRS-1

**Figure 6:**
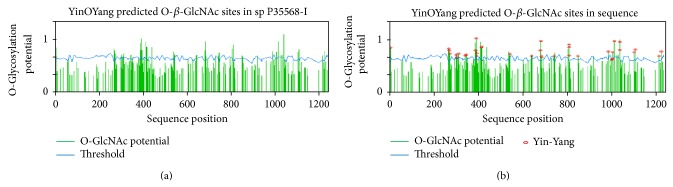
Graphical representation of the potential Ser and Thr residues for O-*β* *-*GlcNAc as well as yin yang sites in human IRS-1. (a) Predicted potential sites for O-*β* *-*GlcNAc modification of Ser and Thr. Green vertical line shows O-*β*-GlcNAc modification potential of Ser/Thr residues, while the light blue wavy line indicates the threshold for modification potential. YinOYang 1.2 web based program generated this graph. A total number of 57 potential Ser/Thr residues showed O-*β*-GlcNAc modification potential including Ser-7, 268, 270, 273, 295, 303, 312, 341, 348, 388, 391, 393, 394, 395, 412, 413, 417, 440, 541, 543, 624, 639, 641, 680, 681, 682, 683, 684, 741, 807, 808, 809, 917, 985, 1000, 1005, 1011, 1025, 1035, 1036, 1037, 1101, 1105, 1213, and 1223 and Thr-305, 311, 351, 387, 539, 743, 803, 847, 851, 1004, 1030, and 1045. (b) Predicted yin yang residues for human IRS-1. A total of 37 Ser/Thr residues showed potential for possible yin yang sites for phosphorylation and O-glycosylation interplay.

YinOYang prediction server showed that the human IRS-1 has 57 potential sites for O-*β*-GlcNAc modifications (Figure 6(a)). It had been studied by various researchers that phosphorylate Ser/Thr residues can also undergo O-*β*-GlcNAc modification when OGT and kinases compete for same site modification [52–54], thus indicating a possibility for crosstalk between O-glycosylation and phosphorylation on these residues. YinOYang 1.2 showed that IRS-1 has high potential for O-glycosylation/phosphorylation interplay (Table 3) and 37 possible yin yang sites were predicted for the crosstalk of phosphorylation and O*-* *β* *-*GlcNAc modification (Figure 6(b)). These sites are Ser-7, 268, 270, 273, 303, 312, 341, 348, 388, 391, 393, 395, 413, 417, 543, 639, 680, 681, 683, 684, 741, 807, 808, 809, 985, 1000, 1005, 1011, 1035, 1036, 1101, 1105, 1213, and 1223 and Thr-305, 847, and 1004 (Table 3). Out of these 37 sites, 7 sites (Ser-7, 680, 681, 683, 684, 1036, and 1213) were labeled as false positive (FP) yin yang sites because they are nonconserved. Meanwhile, some conserved Ser and Thr residues that were not predicted to be O*-* *β* *-*GlcNAc modified, but exhibited very high phosphorylation potential, and also close to threshold value of O-glycosylation, are named as false negative (FN) yin yang sites. These residues include Ser-383, 412, 1037 and Thr-387.

#### 3.4.7. Prediction of O-*β*-GlcNAc Modification and Potential Yin Yang Sites for IRS-2

**Figure 7:**
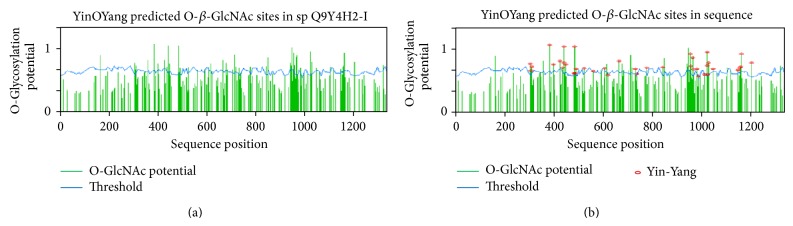
Graphical representation of the potential Ser and Thr residues for O-*β* *-*GlcNAc as well as yin yang sites in human IRS-2. (a) Predicted potential sites for O-*β* *-*GlcNAc modification of Ser and Thr. Green vertical line shows O-*β*-GlcNAc modification potential of Ser/Thr residues, while the light blue wavy line indicates the threshold for modification potential. YinOYang 1.2 web based program generated this graph. A total number of 64 potential Ser/Thr residues showed O-*β*-GlcNAc modification potential including Ser-3, 162, 304, 308, 311, 357, 384, 400, 428, 438, 444, 449, 482, 483, 485, 489, 523, 560, 591, 592, 606, 615, 619, 620, 665, 679, 684, 704, 714, 731, 736, 848, 941, 944, 945, 946, 952, 953, 956, 966, 967, 984, 988, 1012, 1022, 1024, 1026, 1027, 1048, 1103, 1149, 1162, and 1203 and Thr-344, 443, 713, 719, 777, 844, 965, 1157, 1159, 1202, and 1319. (b) Predicted yin yang residues for human IRS-1. A total of 41 Ser/Thr residues showed potential for possible yin yang sites for phosphorylation and O-glycosylation interplay.

According to YinOYang 1.2 results, IRS-2 had high potential for O*-* *β* *-*GlcNAc modification and 64 Ser/Thr residues were predicted to be O-glycosylated (Figure 7(a)). As described earlier, these sites can also undergo phosphorylation modification that may lead to interplay between O-glycosylation and phosphorylation on these sites. YinOYang 1.2 predicted 40 possible yin yang sites (Figure 7(b)). These sites were Ser-304, 308, 311, 384, 400, 428, 438, 444, 449, 482, 483, 485, 489, 523, 560, 606, 620, 665, 731, 736, 953, 956, 967, 984, 988, 1012, 1022, 1024, 1026, 1027, 1048, 1149, 1162, and 1203 and Thr-443, 777, 844, 965, 1157, and 1159 (Table 4). Ser-162, 342, 357, 447, 714, 848, 946, 966, and 1115, and Thr-713 and 1202 were predicted as FN-yin yang sites.

## 4. Discussion

Insulin is a primary hormone that regulates glucose transport and homeostasis in cell systems through its interaction with its receptors (IR) via tyrosine autophosphorylation of IR. Activation of IR subsequently phosphorylates its substrates (IRSs). Insulin receptor substrates 1 and 2 are specific substrates for IR/IGFR and widely expressed in mammalian tissues. Both IRS 1 and 2 contain phosphotyrosine-binding (PTB) domains. Tyrosine phosphorylation of IRS proteins is well studied and more than 20 potential tyrosine phosphorylation sites are mapped on IRSs. During insulin signaling, IRS proteins phosphorylate and activate AKT/PKB/MAPK pathways that regulate insulin mediated metabolic actions and control cell growth and differentiation [10, 20].

Despite Tyr phosphorylation, IRSs also phosphorylate on Ser/Thr residues and phosphorylation on these residues may effect downstream insulin signaling. More than 30 Ser/Thr residues on IRS-1 and 50 on IRS-2 have been mapped using different analytical techniques. However, function of all mapped Ser/Thr residues on IRSs is still undetermined. Our results showed that both IRS-1 and IRS-2 have high potential to be phosphorylated on Ser and Thr residues and more than 100 Ser/Thr residues have been predicted including previously mapped residues on IRS 1 and 2 on both N- and C-terminals including PTB domain (Figures 5(a) and 5(b)). These predicted residues are conserved as shown in Figures 1 and 2 (multiple alignment of IRS-1 and IRS-2, resp.) and may have potential to modify IRSs functioning. Meanwhile various kinases such as GSK3, PKC, PKA, ERK, mTOR, S6K, PKG, CDK5, CKII, and JNK are involved in Ser/Thr phosphorylation of IRSs. Both ERK and mTOR are experimentally verified kinases that can cause phosphorylation at Ser-616 [55]. Phosphorylation of Ser-312 is mediated by increased activation of kinases downstream of PI3-kinase, comprising S6K1, IKK*β*, mTOR, PKC, and JNK [22, 25, 56]. Akt-mTOR activity is highly activated within neurons in many AD cases [57]. S6K activation was also found to be induced in AD that induces phosphorylation of Ser-636 and 639 of IRS-1 [58]. Ser-312, 616, 636, 639, and 1101 in IRS-1 are experimentally verified phosphorylation sites. Phosphorylation of Ser-312 causes IRS-1 proteasomal degradation that leads to insulin resistance under both pathological and physiological conditions, while phosphorylation of Ser-616 is supposed to inhibit insulin activation of PI3-kinase. Furthermore, phosphorylation on Ser-1101 by S6K1 on C-terminal of IRS-1 is thought to be involved in blocking tyrosine as well as AKT phosphorylation that leads to insulin resistance in mice as well as in human [21, 23, 25]. GSK-3 and JNK sequentially phosphorylate IRS-2 at serine residues and its phosphorylation plays inhibitory role in hepatic insulin signaling [51, 58, 59].

The O-*β*-GlcNAc modification is known to be influential and analogous to phosphorylation. Some researchers indicated increased phosphorylation and reduced O-GlcNAc levels in the AD and diabetes patients [60–62]. These findings made O-glycosylation as important PTM in elucidating the mechanism of such metabolic disorders. In this study we used YinOYang 1.2 server to predict O-glycosylation on Ser/Thr residues of both IRS-1 and 2, and more than 50 Ser/Thr residues were shown to have high potential for O-*β*-GlcNAc modification on IRS-1 (Figures 6(a) and 7(a) and Tables 3 and 4). O-*β*-GlcNAc modification in human IRS-1 has been reported at Ser-984 or 985, 1011, and possibly at multiple residues within 1025–1045 [33]. Based on our results Ser-984 is more prone to be O-glycosylated than Ser-985 while Ser-1030, 1035, 1037 and Thr-1045 have potential to be O-glycosylated in 1025–1045 sequence on the basis of conservation. Meanwhile western blotting and site-directed mutagenesis studies in rat suggested Ser-1036 (human numbering Ser-1037) on IRS-1 as possible site of O-*β*-GlcNAc modification. Ser-1037 is conserved in human IRS-1 and identification of this site will be helpful in exploring the biological implication of the O-*β*-GlcNAc modification [33]. Although to date no O-glycosylated residue is mapped on IRS-2, experimental data showed that IRS-2 also have potential to be glycosylated as IRS-1 and this glycosylation was shown to be reduced in diabetes and AD [32]. In this study we have predicted more than 65 Ser/Thr residues on IRS-2 that can act as potential O-glycosylated sites and may ease researchers to map correct O-glycosylated residues on IRS-2.

**Figure 8:**
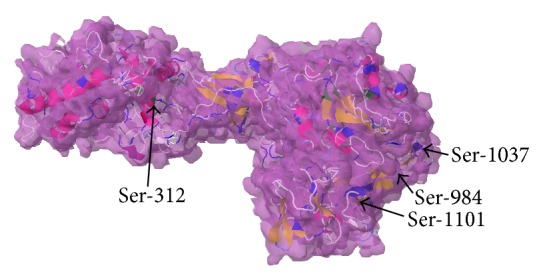
Space filled model of human IRS-1 protein showing higher surface accessibility of possible therapeutic yin yang residues for interplay between phosphorylation and O-glycosylation. Blue and green colours denote the Ser and Thr residues, respectively.

It is established fact that O*-* *β* *-*GlcNAc can mimic or inhibit phosphorylation on the neighboring or same residues and if this interplay happened on same Ser/Thr residues these sites are called yin yang residues. These yin yang residues can be used as therapeutic targets to overcome different diseases [42, 52]. Protein 3D structures are best models to tell whether the predicted residues are accessible or not for these types of interplays [27, 41]. However, for both IRS-1 and IRS-2, complete 3D have not been determined yet, so we used ab initio models to design the complete 3D structures of both proteins. As both IRS-1 and IRS-2 are involved in insulin signaling mechanism, best possible 3D structures for both proteins were selected on basis of Ramachandran plots (Figures 3(a) and 3(b)) and were aligned together to be compared with each other (Figure 4). We found that both proteins have significantly nonidentical structures as their secondary structure previously described. To determine yin yang sites and surface accessibility, we predicted surface accessible residues on the basis of their secondary structure by using NetPhos P server (as given in Table S1 and S2 for both IRS-1 and IRS-2, resp.). Secondary structure based prediction method showed that both proteins have high accessibility for PTMs on Ser/Thr residues except Ser-268, 273, 312, 393, 394, 440, 1223 on IRS-1, Ser-428, and Thr-344. Although secondary structure based prediction method showed no/reduced surface accessibility for Ser-312 of IRS-1, predicted 3D structure revealed that Ser-312 is accessible for PTMs (Figure 8). Moreover, Ser-312 is experimentally verified and known phosphorylation residue, which confirms the exposed accessibility of this residue. Ser-312 phosphorylation is important in insulin resistance and has negative regulation of insulin signaling. Interestingly, Ser-312 is situated very near to PTB domain (Figure 8) and any change in its vicinity via PTMs should alter surrounding areas [21]. Furthermore phosphorylation on three important Ser residues 984, 1037, and 1101 in C-terminal of IRS-1 also has been shown to induce insulin resistance [33]. All these residues were conserved, accessible for such modifications based on 3D structure, and predicted as yin yang residues in our study. This finding showed that alternative O-glycosylation on these residues can inhibit the protein functioning rise by phosphorylation. Although a lot of phosphorylation sites mapped on IRS-2 like Ser-303, 343, 675, and 907 are thought to be involved in inducing insulin resistance, we were unable to predict O-glycosylation on these residues due to limited experimental data availability. On the other hand, we found more than 40 Ser/Thr residues on IRS-2 that has high potential to act as possible yin yang sites (Table 4), and many of them may act as therapeutic targets in future.

Here we propose that alternative O-glycosylation on Ser-312 at N-terminal and Ser-984, Ser-1037, and Ser-1101 at C-terminal of IRS-1 is an important modification that becomes reduced in diabetes and AD due to impaired glucose metabolism. During normal circumstances, O-glycosylation can block phosphorylation on these residues and can act as possible therapeutic sites to reduce risk of diabetes and AD.

## Supplementary Material

Table S1 provides detailed analysis about experimental and predicted phosphorylation, kinases involved and surface accessibility for Ser/ Thr/ Tyr residues of human IRS-1.

## Figures and Tables

**Table 1:** Different IRS-1 proteins used for multiple alignment.

Species name	Accession number	Identity	Score	*E*-value
Human (*Homo sapiens*)	P35568	100%	6,593	0.0
Green monkey (*Chlorocebus aethiops*)	Q28224	96%	6,392	0.0
Pig (*Sus scrofa*)	Q68H99	92%	6,016	0.0
Chicken (*Gallus gallus*)	P79773	90%	5,854	0.0
Chinese hamster (*Cricetulus griseus*)	G3IDC4	89%	5,804	0.0
Mouse (*Mus musculus*)	Q543V3	89%	5,786	0.0
Rat (*Rattus norvegicus*)	P35570	89%	5,783	0.0
Western clawed frog (*Xenopus tropicalis*)	F6XDD6	59%	3,161	0.0

**Table 2:** Different IRS-2 proteins used for multiple alignment.

Species name	Accession number	Identity	Score	*E*-value
Human (*Homo sapiens*)	Q9Y4H2	100%	7,108	0.0
Rat (*Rattus norvegicus*)	F1MAL5	85%	5,908	0.0
Mouse (*Mus musculus*)	P81122	85%	5,891	0.0

**Table 3:** Experimental and predicted O-*β*-GlcNAc and yin yang sites in human IRS-1.

Substrate			O-*β*-GlcNAc status		Yin yang residues
Residues	PS	Conservation	SA	YinOYang 1.2	
Ser	7	NC	E	++	Y(FP)
Ser	268	CS	B	++	Y
Ser	270	C	E	++	Y
Ser	273	C	B	+	Y
Ser	295	C	E	+	−
Ser	303	CS	E	+	Y
Thr	305	C	E	+	Y
Thr	311	C	E	+	−
Ser	312	C	B	+	Y
Ser	341	SC	E	+	Y
Ser	348	C	E	+	Y
Thr	351	CS	E	+	−
Ser	383	C	E	−	Y(FN)
Thr	387	C	E	+++	Y(FN)
Ser	388	C	E	++	Y
Ser	391	C	E	++++	Y
Ser	393	C	B	+	Y
Ser	394	C	B	+	−
Ser	395	C	E	+	Y
Ser	412	C	E	+++	Y(FN)
Ser	413	C	E	++	Y
Ser	417	C	E	++	Y
Ser	440	C	B	++	−
Thr	539	C	E	++	−
Ser	541	C	E	+	−
Ser	543	C	E	+	Y
Ser	624	NC	E	+	−
Ser	639	C	E	+	Y
Ser	641	C	E	+	−
Ser	680	NC	E	++	Y(FP)
Ser	681	NC	E	+++	Y(FP)
Ser	682	NC	E	++	−
Ser	683	NC	E	+	Y(FP)
Ser	684	NC	E	+	Y(FP)
Ser	741	C	E	+	Y
Thr	743	C	E	++	−
Thr	803	NC	E	++	−
Ser	807	C	E	+	Y
Ser	808	C	E	+++	Y
Ser	809	C	E	++	Y
Thr	847	C	E	+	Y
Thr	851	NC	E	+	−
Ser	917	NC	E	+	−
Ser	985	SC	E	+	Y
Ser	1000	SC	E	+	Y
Thr	1004	SC	E	+	Y
Ser	1005	SC	E	+	Y
Ser	1011	CS	E	++	Y
Ser	1025	SC	E	+	−
Thr	1030	SC	E	+	−
Ser	1035	SC	E	+	Y
Ser	1036	NC	E	+++	Y(FP)
Ser	1037	CS	E	++++	Y(FN)
Thr	1045	NC	E	+	−
Ser	1101	C	E	+	Y
Ser	1105	C	E	++	Y
Ser	1213	NC	E	+	Y(FP)
Ser	1223	C	B	++	Y

PS: position; SA: surface accessibility; E: exposed; B, buried; C: conserved; NC: nonconserved; CS: conserved substitutions; SC: semiconserved; Y: predicted as yin yang sites by YinOYang 1.2; FP: false positive; FN: false negative; −: negative/not determined yet; +: positive; ++: highly positive; +++: very highly positive; ++++: very very highly positive.

**Table 4:** Experimental and predicted O-*β*-GlcNAc and yin yang sites in human IRS-2.

Substrate			O-*β*-GlcNAc status		Yin yang residues
Residues	PS	Conservation	SA	YinOYang 1.2	
Ser	3	C	E	+	−
Ser	162	C	E	++	Y(FN)
Ser	304	C	E	++	Y
Ser	308	C	E	+	Y
Ser	311	C	E	+	Y
Ser	342	C	E	−	Y(FN)
Thr	344	C	B	+	−
Ser	357	CS	E	++	Y(FN)
Ser	384	C	E	++++	Y
Ser	400	C	E	+	Y
Ser	428	C	B	+	Y
Ser	438	C	E	+	Y
Thr	443	C	E	++++	Y
Ser	444	C	E	++	Y
Ser	447	C	E	−	Y(FN)
Ser	449	C	E	+	Y
Ser	482	C	E	+	Y
Ser	483	C	E	++++	Y
Ser	485	C	E	+	Y
Ser	489	C	E	+	Y
Ser	523	C	E	+	Y
Ser	560	C	E	+	Y
Ser	591	C	E	+	−
Ser	592	C	E	+	−
Ser	606	C	E	+	Y
Ser	615	C	E	+	−
Ser	619	C	E	+	−
Ser	620	C	E	+	Y
Ser	665	SC	E	++	Y
Ser	679	C	E	+	−
Ser	684	C	E	+	−
Ser	704	SC	E	+	−
Thr	713	SC	E	++	Y(FN)
Ser	714	C	E	++	Y(FN)
Thr	719	C	E	+	−
Ser	731	C	E	+	Y
Ser	736	C	E	+	Y
Thr	777	CS	E	+	Y
Thr	844	C	E	++	Y
Ser	848	CS	E	++	Y(FN)
Ser	941	C	E	+	−
Ser	944	C	E	+	−
Ser	945	C	E	+	−
Ser	946	C	E	++++	Y(FN)
Ser	952	C	E	+	−
Ser	953	C	E	++	Y
Ser	956	C	E	+	Y
Thr	965	C	E	++	Y
Ser	966	C	E	+++	Y(FN)
Ser	967	C	E	+	Y
Ser	984	C	E	+	Y
Ser	988	C	E	+	Y
Ser	1012	C	E	+	Y
Ser	1022	C	E	+++	Y
Ser	1024	C	E	++	Y
Ser	1026	C	E	+	Y
Ser	1027	C	E	++	Y
Ser	1048	CS	E	+	Y
Ser	1103	C	E	+	−
Ser	1115	C	E	−	Y(FN)
Ser	1149	C	E	+	Y
Thr	1157	C	E	+	Y
Thr	1159	C	E	+	Y
Ser	1162	C	E	++	Y
Thr	1202	C	E	++	Y(FN)
Ser	1203	C	E	++	Y
Thr	1319	C	B	+	−

PS: position; SA: surface accessibility; E: exposed; B: buried; C: conserved; CS: conserved substitutions; SC: semiconserved; Y: predicted as yin yang sites by YinOYang 1.2; FN: false negative; −: negative/not determined yet; +: positive; ++: highly positive; +++: very highly positive; ++++: very very highly positive.

## References

[1] Selkoe D. J. (1997). Alzheimer's disease: genotypes, phenotype, and treatments. *Science*.

[2] Alzheimer's Association (2012). 2012 Alzheimer's disease facts and figures. *Alzheimer's & Dementia*.

[3] Gispen W. H., Biessels G.-J. (2000). Cognition and synaptic plasticity in diabetes mellitus. *Trends in Neurosciences*.

[4] Hoyer S. (1998). Is sporadic Alzheimer disease the brain type of non-insulin dependent diabetes mellitus? A challenging hypothesis. *Journal of Neural Transmission*.

[5] de la Monte S. M., Wands J. R. (2008). Alzheimer's disease is type 3 diabetes-evidence reviewed. *Journal of Diabetes Science and Technology*.

[6] Ahmad W. (2013). Overlapped metabolic and therapeutic links between Alzheimer and diabetes. *Molecular Neurobiology*.

[7] Sun M.-K., Alkon D. L. (2006). Links between Alzheimer's disease and diabetes. *Drugs of Today*.

[8] Deng Y., Li B., Liu Y., Iqbal K., Grundke-Iqbal I., Gong C.-X. (2009). Dysregulation of insulin signaling, glucose transporters, O-GlcNAcylation, and phosphorylation of tau and neurofilaments in the brain: implication for Alzheimer's disease. *The American Journal of Pathology*.

[9] Ke Y. D., Delerue F., Gladbach A., Götz J., Ittner L. M. (2009). Experimental diabetes mellitus exacerbates Tau pathology in a transgenic mouse model of Alzheimer's disease. *PLoS ONE*.

[10] Sesti G., Federici M., Hribal M. L., Lauro D., Sbraccia P., Lauro R. (2001). Defects of the insulin receptor substrate (IRS) system in human metabolic disorders. *FASEB Journal*.

[11] Withers D. J., Burks D. J., Towery H. H., Altamuro S. L., Flint C. L., White M. F. (1999). Irs-2 coordinates Igf-1 receptor-mediated *β*-cell development and peripheral insulin signalling. *Nature Genetics*.

[12] Withers D. J., Gutierrez J. S., Towery H., Burks D. J., Ren J.-M., Previs S., Zhang Y., Bernal D., Pons S., Shulman G. I., Bonner-Weir S., White M. F. (1998). Disruption of IRS-2 causes type 2 diabetes in mice. *Nature*.

[13] Selman C., Lingard S., Choudhury A. I., Batterham R. L., Claret M., Clements M., Ramadani F., Okkenhaug K., Schuster E., Blanc E., Piper M. D., Al-Qassab H., Speakman J. R., Carmignac D., Robinson I. C. A., Thornton J. M., Gems D., Partridge L., Withers D. J. (2008). Evidence for lifespan extension and delayed age-related biomarkers in insulin receptor substrate 1 null mice. *FASEB Journal*.

[14] Taguchi A., Wartschow L. M., White M. F. (2007). Brain IRS2 signaling coordinates life span and nutrient homeostasis. *Science*.

[15] Schubert M., Brazil D. P., Burks D. J., Kushner J. A., Ye J., Flint C. L., Farhang-Fallah J., Dikkes P., Warot X. M., Rio C., Corfas G., White M. F. (2003). Insulin receptor substrate-2 deficiency impairs brain growth and promotes tau phosphorylation. *The Journal of Neuroscience*.

[16] Moloney A. M., Griffin R. J., Timmons S., O'Connor R., Ravid R., O'Neill C. (2010). Defects in IGF-1 receptor, insulin receptor and IRS-1/2 in Alzheimer's disease indicate possible resistance to IGF-1 and insulin signalling. *Neurobiology of Aging*.

[17] Dearth R. K., Cui X., Kim H.-J., Hadsell D. L., Lee A. V. (2007). Oncogenic transformation by the signaling adaptor proteins insulin receptor substrate (IRS)-1 and IRS-2. *Cell Cycle*.

[18] Mann M., Jensen O. N. (2003). Proteomic analysis of post-translational modifications. *Nature Biotechnology*.

[19] Summers S. A. (2006). Ceramides in insulin resistance and lipotoxicity. *Progress in Lipid Research*.

[20] Sesti G. (2006). Pathophysiology of insulin resistance. *Best Practice and Research: Clinical Endocrinology and Metabolism*.

[21] Greene M. W., Sakaue H., Wang L., Alessi D. R., Roth R. A. (2003). Modulation of insulin-stimulated degradation of human insulin receptor substrate-1 by serine 312 phosphorylation. *The Journal of Biological Chemistry*.

[22] Griffin R. J., Moloney A., Kelliher M., Johnston J. A., Ravid R., Dockery P., O'Connor R., O'Neill C. (2005). Activation of Akt/PKB, increased phosphorylation of Akt substrates and loss and altered distribution of Akt and PTEN are features of Alzheimer's disease pathology. *Journal of Neurochemistry*.

[23] Rui L., Aguirre V., Kim J. K., Shulman G. I., Lee A., Corbould A., Dunaif A., White M. F. (2001). Insulin/IGF-1 and TNF-*α* stimulate phosphorylation of IRS-1 at inhibitory Ser^307^ via distinct pathways. *The Journal of Clinical Investigation*.

[24] Zick Y. (2005). Ser/Thr phosphorylation of IRS proteins: a molecular basis for insulin resistance. *Science's STKE*.

[25] Yu H., Rohan T. (2000). Role of the insulin-like growth factor family in cancer development and progression. *Journal of the National Cancer Institute*.

[26] Firth S. M., Baxter R. C. (2002). Cellular actions of the insulin-like growth factor binding proteins. *Endocrine Reviews*.

[27] Ahmad W., Shabbiri K., Ijaz B., Asad S., Nazar N., Nazar S., Fouzia K., Kausar H., Gull S., Sarwar M. T., Shahid I., Hassan S. (2011). Serine 204 phosphorylation and O - GlcNAC interplay of IGFBP-6 as therapeutic indicator to regulate IGF-II functions in viral mediated hepatocellular carcinoma. *Virology Journal*.

[28] Wu C. H., Apweiler R., Bairoch A. (2006). The Universal Protein Resource (UniProt): an expanding universe of protein information. *Nucleic Acids Research*.

[29] Zachara N. E., Hart G. W. (2002). The emerging significance of O-GlcNAc in cellular regulation. *Chemical Reviews*.

[30] White M. F. (2006). Regulating insulin signaling and *β*-cell function through IRS proteins. *Canadian Journal of Physiology and Pharmacology*.

[31] Petersen K. F., Shulman G. I. (2006). Etiology of insulin resistance. *American Journal of Medicine*.

[32] D'Alessandris C., Andreozzi F., Federici M., Cardellini M., Brunetti A., Ranalli M., Del Guerra S., Lauro D., Del Prato S., Marchetti P., Lauro R., Sesti G. (2004). Increased *O*-glycosylation of insulin signaling proteins results in their impaired activation and enhanced susceptibility to apoptosis in pancreatic *β*-cells. *The FASEB Journal*.

[33] Kleini A. L., Berkaw M. N., Buse M. G., Ball L. E. (2009). O-linked N-acetylglucosamine modification of insulin receptor substrate-1 occurs in close proximity to multiple SH2 domain binding motifs. *Molecular and Cellular Proteomics*.

[34] Ball L. E., Berkaw M. N., Buse M. G. (2006). Identification of the major site of O-linked *β*-N-acetylglucosamine modification in the C terminus of insulin receptor substrate-1. *Molecular and Cellular Proteomics*.

[35] Eswar N., Webb B., Marti-Renom M. A., Madhusudhan M. S., Eramian D., Shen M. Y., Pieper U., Sali A. (2006). Unit 5.6. Chapter 5. Comparative protein structure modeling using modeller. *Current Protocols in Bioinformatics*.

[36] Zhang Y. (2008). I-TASSER server for protein 3D structure prediction. *BMC Bioinformatics*.

[37] Arnold K., Bordoli L., Kopp J., Schwede T. (2006). The SWISS-MODEL workspace: a web-based environment for protein structure homology modelling. *Bioinformatics*.

[38] Davis I. W., Murray L. W., Richardson J. S., Richardson D. C. (2004). MolProbity: structure validation and all-atom contact analysis for nucleic acids and their complexes. *Nucleic Acids Research*.

[39] Pettersen E. F., Goddard T. D., Huang C. C. (2004). UCSF Chimera—a visualization system for exploratory research and analysis. *Journal of Computational Chemistry*.

[40] Baldi P., Brunak S. (2001). *Bioinformatics: The Machine Learning Approach*.

[41] Ahmad W., Shabbiri K., Ijaz B., Asad S., Sarwar M. T., Gull S., Kausar H., Fouzia K., Shahid I., Hassan S. (2011). Claudin-1 required for HCV virus entry has high potential for phosphorylation and O-glycosylation. *Virology Journal*.

[42] Ahmad W., Shabbiri K., Nazar N., Nazar S., Qaiser S., Mughal M. A. S. (2011). Human linker histones: interplay between phosphorylation and O-*β*-GlcNAc to mediate chromatin structural modifications. *Cell Division*.

[43] Blom N., Gammeltoft S., Brunak S. (1999). Sequence and structure-based prediction of eukaryotic protein phosphorylation sites. *Journal of Molecular Biology*.

[44] Songyang Z., Blechner S., Hoagland N., Hoekstra M. F., Piwnica-Worms H., Cantley L. C. (1994). Use of an oriented peptide library to determine the optimal substrates of protein kinases. *Current Biology*.

[45] Blom N., Sicheritz-Pontén T., Gupta R., Gammeltoft S., Brunak S. (2004). Prediction of post-translational glycosylation and phosphorylation of proteins from the amino acid sequence. *Proteomics*.

[46] Huang H.-D., Lee T.-Y., Tzeng S.-W., Horng J.-T. (2005). KinasePhos: a web tool for identifying protein kinase-specific phosphorylation sites. *Nucleic Acids Research*.

[47] Diella F., Cameron S., Gemünd C., Linding R., Via A., Kuster B., Sicheritz-Pontén T., Blom N., Gibson T. J. (2004). Phospho.ELM: a database of experimentally verified phosphorylation sites in eukaryotic proteins. *BMC Bioinformatics*.

[48] Gupta R., Brunak S. (2002). Prediction of glycosylation across the human proteome and the correlation to protein function. *Pacific Symposium on Biocomputing*.

[49] Petersen B., Petersen T. N., Andersen P., Nielsen M., Lundegaard C. (2009). A generic method for assignment of reliability scores applied to solvent accessibility predictions. *BMC Structural Biology*.

[50] Obenauer J. C., Cantley L. C., Yaffe M. B. (2003). Scansite 2.0: proteome-wide prediction of cell signalling interactions using short sequence motifs. *Nucleic Acids Research*.

[51] Sharfi H., Eldar-Finkelman H. (2008). Sequential phosphorylation of insulin receptor substrate-2 by glycogen synthase kinase-3 and c-Jun NH2-terminal kinase plays a role in hepatic insulin signaling. *American Journal of Physiology: Endocrinology and Metabolism*.

[52] Ahmad I., Hoessli D. C., Walker-Nasir E., Rafik S. M., Shakoori A. R. (2006). Oct-2 DNA binding transcription factor: functional consequences of phosphorylation and glycosylation. *Nucleic Acids Research*.

[53] Kaleem A., Hoessli D. C., Ahmad I., Walker-Nasir E., Nasim A., Shakoori A. R., Nasir-ud D. (2008). Immediate-early gene regulation by interplay between different post-translational modifications on human histone H3. *Journal of Cellular Biochemistry*.

[54] Kaleem A., Hoessli D. C., Haq I.-U. (2011). CREB in long-term potentiation in hippocampus: role of post-translational modifications-studies in silico. *Journal of Cellular Biochemistry*.

[55] Gao Z., Hwang D., Bataille F., Lefevre M., York D., Quon M. J., Ye J. (2002). Serine phosphorylation of insulin receptor substrate 1 by inhibitor *κ*B kinase complex. *Journal of Biological Chemistry*.

[56] Haruta T., Uno T., Kawahara J., Takano A., Egawa K., Sharma P. M., Olefsky J. M., Kobayashi M. (2000). A rapamycin-sensitive pathway down-regulates insulin signaling via phosphorylation and proteasomal degradation of insulin receptor substrate-1. *Molecular Endocrinology*.

[57] An W.-L., Cowburn R. F., Li L., Braak H., Alafuzoff I., Iqbal K., Iqbal I.-G., Winblad B., Pei J.-J. (2003). Up-regulation of phosphorylated/activated p70 S6 kinase and its relationship to neurofibrillary pathology in Alzheimer's disease. *The American Journal of Pathology*.

[58] Neukamm S. S., Toth R., Morrice N., Campbell D. G., MacKintosh C., Lehmann R., Haering H.-U., Schleicher E. D., Weigert C. (2012). Identification of the Amino acids 300-600 of IRS-2 as 14-3-3 binding region with the importance of IGF-1/insulin-regulated phosphorylation of Ser-573. *PLoS ONE*.

[59] Zhande R., Zhang W., Zheng Y. (2006). Dephosphorylation by default, a potential mechanism for regulation of insulin receptor substrate-1/2, Akt, and ERK1/2. *The Journal of Biological Chemistry*.

[60] Liu F., Shi J., Tanimukai H., Gu J., Gu J., Grundke-Iqbal I., Iqbal K., Gong C.-X. (2009). Reduced O-GlcNAcylation links lower brain glucose metabolism and tau pathology in Alzheimer's disease. *Brain*.

[61] Yu Y., Zhang L., Li X., Run X., Liang Z., Li Y., Liu Y., Lee M. H., Grundke-Iqbal I., Iqbal K., Vocadlo D. J., Liu F., Gong C.-X. (2012). Differential effects of an O-GlcNAcase inhibitor on tau phosphorylation. *PLoS ONE*.

[62] Yuzwa S. A., Shan X., MacAuley M. S., Clark T., Skorobogatko Y., Vosseller K., Vocadlo D. J. (2012). Increasing O-GlcNAc slows neurodegeneration and stabilizes tau against aggregation. *Nature Chemical Biology*.

