# Supplementary material for: Alternate Phosphorylation/O-GlcNAc Modification on Human Insulin IRSs: A Road towards Impaired Insulin Signaling in Alzheimer and Diabetes
Source: Adv Bioinformatics. 2014 Dec 17;2014:324753. doi: 10.1155/2014/324753 (PMC4281456; doi:10.1155/2014/324753)
Supplement: Supplementary file 1 — Table S1 provides detailed analysis about experimental and predicted phosphorylation, kinases involved and surface accessibility for Ser/ Thr/ Tyr residues of human IRS-1. [file 324753.f1.doc]

| **Table S1**  **Experimental and predicted phosphorylation, kinases involved and surface accessibility for Ser/ Thr/ Tyr residues of human IRS-1. Abbreviation used in table: PS, position; EV, experimentally verified; C, conserved; CS, conserved substitutions; SC, semi-conserved; NC, non-conserved; Y, yes (threshold > 0.5) (predicted or experimentally verified phosphorylated residues); P, probable (threshold = 0.1~0.5) (may be phosphorylated residues); N, No (threshold < 0.1) (not predicted or not experimentally verified phosphorylated residues); E, exposed surface; B, buried surface; >1, high surface accessibility; <1, low surface accessibility, 1 = low potential for solvent accessibility, 2 = high potential for solvent accessibility.** | | | | | | | | | | | |
| --- | --- | --- | --- | --- | --- | --- | --- | --- | --- | --- | --- |
| **Substrate** | | | **Phosphorylation** | | |  | **Kinases Involved** | | | **Surface accessibility** | |
| **Residue** | **PS** | **Cons.** | **EV** | **Netphos** | **Scansite** | **Kinasephos**  **(Score)** | **NetphosK** | **Scansite** | **Kinasephos** | **Scansite** | **NetSurfP** |
| Serine | 3 | NC | N | Y | - | - | p38MAPK,GSK3, cdk5 | - | - | - | E |
|  | 7 | NC | N | Y | - | - | GSK3 | - | - | - | E |
|  | 11 | C | N | Y | - | - | - | - | - | - | E |
|  | 24 | C | Y | Y | P | - | PKC | PKC epsilon, PKC zeta, PKC alpha/beta/gamma, PKC mu | - | >1 | E |
|  | 36 | C | N | Y | - | -4.2 | - | - | [PKG](http://kinasephos.mbc.nctu.edu.tw/help.html#kinase) | - | B |
|  | 57 | C | N | P | - | - | RSK, PKA, PKG | - | - | - | E |
|  | 58 | SC | N | N | P | - | PKC | PKC alpha/beta/gamma | - | >1 | E |
|  | 63 | C | N | N | - | - | PKA | - | - | - | B |
|  | 68 | C | N | N | - | - | - | - | - | - | E |
|  | 78 | SC | N | Y | - | -5.4 | RSK, PKC, PKA | - | [PKG](http://kinasephos.mbc.nctu.edu.tw/help.html#kinase) | - | B |
|  | 99 | SC | N | Y | - | 3.4 | CKII | - | [CKII](http://kinasephos.mbc.nctu.edu.tw/help.html#kinase) | - | E |
|  | 105 | SC | N | Y | - | - | - | - | - | - | E |
|  | 135 | SC | N | P | - | - | - | - | - | - | B |
|  | 137 | CS | N | P | - | -1.0 | - | - | [ATM](http://kinasephos.mbc.nctu.edu.tw/help.html#kinase) | - | E |
|  | 139 | SC | N | P | - | - | - | - | - | - | E |
|  | 140 | NC | N | P | - | - | cdc2 | - | - | - | E |
|  | 150 | NC | N | Y | - | - | CKI | - | - | - | E |
|  | 189 | SC | N | P | - | - | - | - | - | - | E |
|  | 193 | C | N | P | - | - | - | - | - | - | B |
|  | 199 | C | N | P | - | - | - | - | - | - | B |
|  | 217 | C | N | Y | - | - | - | - | - | - | E |
|  | 228 | C | N | Y | - | - | - | - | - | - | E |
|  | 243 | C | N | Y | - | - | - | - | - | - | B |
|  | 261 | C | N | Y | - | - | cdc2 | - | - | - | E |
|  | 268 | CS | Y | Y | - | -0.7 | - | - | [IKK](http://kinasephos.mbc.nctu.edu.tw/help.html#kinase) | - | B |
|  | 270 | C | Y | Y | P | -5.5, 3.2 | RSK, DNAPK, PKB, cdc2 | 14-3-3 Mode 1, Akt Kinase | [PKG](http://kinasephos.mbc.nctu.edu.tw/help.html#kinase), [IKK](http://kinasephos.mbc.nctu.edu.tw/help.html#kinase) | >1 | E |
|  | 272 | C | Y | Y | - | -2.3 | - | - | [IKK](http://kinasephos.mbc.nctu.edu.tw/help.html#kinase) | - | E |
|  | 273 | C | N | Y | - | - | cdc2 | - | - | - | B |
|  | 274 | C | Y | Y | - | 2.4 | PKC, cdc2 | - | [IKK](http://kinasephos.mbc.nctu.edu.tw/help.html#kinase) | - | E |
|  | 277 | C | N | N | - | -3.7 | - | - | [ATM](http://kinasephos.mbc.nctu.edu.tw/help.html#kinase) | - | B |
|  | 281 | C | N | N | - | - | GSK3 | - | - | - | E |
|  | 295 | C | N | P | - | - | DNAPK | - | - | - | E |
|  | 303 | CS | N | Y | - | 3.6, -4.3 | PKC, PKA, PKG, cdc2 | - | [PKA](http://kinasephos.mbc.nctu.edu.tw/help.html#kinase), [PKG](http://kinasephos.mbc.nctu.edu.tw/help.html#kinase) | - | E |
|  | 307 | C | Y | Y | P | -5.5, 1.8, 0.8 | RSK,PKB, PKC | Akt Kinase, PKC delta, PKC epsilon | [PKG](http://kinasephos.mbc.nctu.edu.tw/help.html#kinase), [IKK](http://kinasephos.mbc.nctu.edu.tw/help.html#kinase), [PKB](http://kinasephos.mbc.nctu.edu.tw/help.html#kinase) | <1 | E |
|  | 312 | C | Y | Y | - | -3.3, -1.6, 3.1 | GSK3, cdk5 | - | [cdc2](http://kinasephos.mbc.nctu.edu.tw/help.html#kinase), [IKK](http://kinasephos.mbc.nctu.edu.tw/help.html#kinase), [Other_MDD](http://kinasephos.mbc.nctu.edu.tw/help.html#kinase) | - | B |
|  | 315 | SC | N | Y | - | - | - | - | - | - | E |
|  | 323 | C | Y | P | - | - | PKC | - | - | - | E |
|  | 329 | C | N | Y | - | 0.0, -2.5 | PKA | - | [CKII](http://kinasephos.mbc.nctu.edu.tw/help.html#kinase), [PKG](http://kinasephos.mbc.nctu.edu.tw/help.html#kinase) | - | E |
|  | 330 | C | N | Y | P | - | CKII, RSK, CKI, PKB, PKA, cdc2 | 14-3-3 Mode 1, Akt Kinase | - | >1 | E |
|  | 337 | C | N | P | - | - | - | - | - | - | E |
|  | 341 | SC | Y | Y | - | 1.6, 1.5 | RSK, PKC | - | [CaM-II](http://kinasephos.mbc.nctu.edu.tw/help.html#kinase), [IKK](http://kinasephos.mbc.nctu.edu.tw/help.html#kinase) | - | E |
|  | 345 | C | Y | Y | - | -1.5, -3.7, 1.7, 3.6 | cdc2, GSK3,cdk5 | - | [cdc2](http://kinasephos.mbc.nctu.edu.tw/help.html#kinase), [ATM](http://kinasephos.mbc.nctu.edu.tw/help.html#kinase), [IKK](http://kinasephos.mbc.nctu.edu.tw/help.html#kinase), [Other_MDD](http://kinasephos.mbc.nctu.edu.tw/help.html#kinase) | - | E |
|  | 348 | C | Y | Y | - | -2.3, -4.2, -0.1 | CKI, p38MAPK, GSK3, cdk5 | - | [cdc2](http://kinasephos.mbc.nctu.edu.tw/help.html#kinase), [ATM](http://kinasephos.mbc.nctu.edu.tw/help.html#kinase), [IKK](http://kinasephos.mbc.nctu.edu.tw/help.html#kinase) | - | E |
|  | 350 | C | N | Y | - | - | PKC, cdc2 | - | - | - | E |
|  | 362 | C | N | Y | - | 5.5 | PKC, PKA | - | [PKA](http://kinasephos.mbc.nctu.edu.tw/help.html#kinase) | - | E |
|  | 372 | C | N | N | - | - | cdc2 | - | - | - | E |
|  | 374 | C | Y | P | - | - | - | - | - | - | E |
|  | 380 | CS | N | P | - | - | cdc2 | - | - | - | B |
|  | 383 | C | N | Y | - | 0.3 | GSK3, cdk5 | - | [MAPK](http://kinasephos.mbc.nctu.edu.tw/help.html#kinase) | - | E |
|  | 385 | C | N | Y | - | - | - | - | - | - | E |
|  | 388 | C | N | Y | - | -0.8, 2.5, -1.7 | cdc2, cdk5 | - | [cdc2](http://kinasephos.mbc.nctu.edu.tw/help.html#kinase), [MAPK](http://kinasephos.mbc.nctu.edu.tw/help.html#kinase), [IKK](http://kinasephos.mbc.nctu.edu.tw/help.html#kinase) | - | E |
|  | 391 | C | N | Y | - | -0.8 | - | - | [IKK](http://kinasephos.mbc.nctu.edu.tw/help.html#kinase) | - | E |
|  | 393 | C | N | Y | - | -0.2, -1.7 | cdc2 | - | [CKII](http://kinasephos.mbc.nctu.edu.tw/help.html#kinase), [IKK](http://kinasephos.mbc.nctu.edu.tw/help.html#kinase) | - | B |
|  | 394 | C | N | P | P | - | CKI | GSK3-improved | - | <1 | B |
|  | 395 | C | N | Y | - | -1.9 | cdc2 | - | [CKI](http://kinasephos.mbc.nctu.edu.tw/help.html#kinase) | - | E |
|  | 396 | C | N | Y | - | -0.5 | cdc2 | - | [CKI](http://kinasephos.mbc.nctu.edu.tw/help.html#kinase) | - | E |
|  | 398 | C | N | Y | P | 1.1, -0.7, -1.9 | CKI | GSK3-improved | [CKII](http://kinasephos.mbc.nctu.edu.tw/help.html#kinase), [CKI](http://kinasephos.mbc.nctu.edu.tw/help.html#kinase), [IKK](http://kinasephos.mbc.nctu.edu.tw/help.html#kinase) | <1 | E |
|  | 402 | C | N | Y | - | - | - | - | - | - | E |
|  | 404 | C | N | P | - | - | CKII, cdc2 | - | - | - | E |
|  | 412 | C | N | N | - | - | PKA | - | - | - | E |
|  | 413 | C | N | Y | P | 0.8, 3.0 | RSK, PKA, PKG | 14-3-3 Mode 1, Protein Kinase A | [PKG](http://kinasephos.mbc.nctu.edu.tw/help.html#kinase), [PKB](http://kinasephos.mbc.nctu.edu.tw/help.html#kinase) | <1 | E |
|  | 415 | C | N | Y | P | 0.6, -0.1 | PKC | PKC delta, GSK3-improved | [CaM-II](http://kinasephos.mbc.nctu.edu.tw/help.html#kinase), [IKK](http://kinasephos.mbc.nctu.edu.tw/help.html#kinase) | <1 | E |
|  | 417 | C | N | Y | P | -3.3, -1.5 | - | GSK3-improved | [ATM](http://kinasephos.mbc.nctu.edu.tw/help.html#kinase), [IKK](http://kinasephos.mbc.nctu.edu.tw/help.html#kinase) | <1 | E |
|  | 419 | C | N | Y | - | -1.4, -2.3, -1.0 | cdc2, GSK3, cdk5 | - | [cdc2](http://kinasephos.mbc.nctu.edu.tw/help.html#kinase), [ATM](http://kinasephos.mbc.nctu.edu.tw/help.html#kinase), [IKK](http://kinasephos.mbc.nctu.edu.tw/help.html#kinase) | - | E |
|  | 421 | C | N | P | - | -3.4 | - | - | [ATM](http://kinasephos.mbc.nctu.edu.tw/help.html#kinase) | - | E |
|  | 427 | C | N | N | - | 1.4 | CKII | - | [CKII](http://kinasephos.mbc.nctu.edu.tw/help.html#kinase) | - | E |
|  | 428 | C | N | P | - | - | CKII, ATM | - | - | - | B |
|  | 433 | C | N | P | - | - | CKII | - | - | - | B |
|  | 434 | C | N | Y | - | - | - | - | - | - | B |
|  | 440 | C | N | P | - | - | - | - | - | - | B |
|  | 441 | C | N | Y | P | - | PKC, cdc2 | PKC alpha/beta/gamma, PKC epsilon | - | >1 | B |
|  | 444 | C | N | Y | - | - | - | - | - | - | E |
|  | 449 | C | N | Y | - | - | - | - | - | - | E |
|  | 463 | SC | N | N | - | - | CKII | - | - | - | B |
|  | 474 | NC | N | P | - | - | cdc2 | - | - | - | E |
|  | 486 | SC | N | N | - | - | PKC | - | - | - | E |
|  | 503 | NC | N | P | - | - | - | - | - | - | E |
|  | 513 | NC | N | Y | - | - | CKII | - | - | - | E |
|  | 527 | C | Y | Y | P | 0.8, -5.7, -1.2 | RSK, PKB, PKA, PKG | 14-3-3 Mode 1, Akt Kinase | [CaM-II](http://kinasephos.mbc.nctu.edu.tw/help.html#kinase), [PKG](http://kinasephos.mbc.nctu.edu.tw/help.html#kinase), [IKK](http://kinasephos.mbc.nctu.edu.tw/help.html#kinase) | <1 | B |
|  | 531 | C | Y | Y | - | -3.0, -1.2 | cdk5 | - | [cdc2](http://kinasephos.mbc.nctu.edu.tw/help.html#kinase), [IKK](http://kinasephos.mbc.nctu.edu.tw/help.html#kinase) | - | E |
|  | 541 | C | N | P | - | -3.8, 3.2 | DNAPK, ATM | - | [ATM](http://kinasephos.mbc.nctu.edu.tw/help.html#kinase), [Other_MDD](http://kinasephos.mbc.nctu.edu.tw/help.html#kinase) | - | E |
|  | 543 | C | N | Y | - | - | - | - | - | - | E |
|  | 544 | NC | N | Y | P | - | PKC | PKC epsilon | - | <1 | E |
|  | 547 | C | N | Y | - | -2.5 | CKII, CKI | - | [CKI](http://kinasephos.mbc.nctu.edu.tw/help.html#kinase) | - | E |
|  | 564 | NC | N | Y | - | - | PKA | - | - | - | B |
|  | 574 | C | Y | Y | - | - | PKA, cdc2 | - | - | - | E |
|  | 581 | C | N | Y | - | - | CKII | - | - | - | E |
|  | 603 | NC | N | Y | - | - | PKA | - | - | - | E |
|  | 604 | NC | N | P | - | - | - | - | - | - | E |
|  | 616 | C | Y | Y | - | - | p38MAPK, GSK3, cdk5 | - | - | - | E |
|  | 624 | NC | N | P | - | - | - | - | - | - | E |
|  | 629 | SC | Y | Y | - | - | RSK, PKA | - | - | - | E |
|  | 636 | C | Y | Y | - | - | GSK3, cdk5 | - | - | - | B |
|  | 639 | C | Y | Y | - | 0.9 | PKC | - | [IKK](http://kinasephos.mbc.nctu.edu.tw/help.html#kinase) | - | E |
|  | 641 | C | N | P | P | -1.7 | - | 14-3-3 Mode 1 | [IKK](http://kinasephos.mbc.nctu.edu.tw/help.html#kinase) | <1 | E |
|  | 666 | C | N | Y | - | - | GSK3 | - | - | - | B |
|  | 668 | C | N | Y | - | - | PKC | - | - | - | E |
|  | 672 | C | N | P | - | -1.7 | GSK3, cdk5 | - | [ATM](http://kinasephos.mbc.nctu.edu.tw/help.html#kinase) | - | E |
|  | 680 | NC | N | Y | - | - | - | - | - | - | E |
|  | 681 | NC | N | Y | - | - | - | - | - | - | E |
|  | 682 | NC | N | P | - | - | cdc2 | - | - | - | E |
|  | 683 | NC | N | Y | - | -2.2 | cdc2 | - | [CKI](http://kinasephos.mbc.nctu.edu.tw/help.html#kinase) | - | E |
|  | 684 | NC | N | Y | - | -2.1 | cdc2 | - | [CKI](http://kinasephos.mbc.nctu.edu.tw/help.html#kinase) | - | E |
|  | 685 | NC | N | Y | - | -1.4 | PKC | - | [CKI](http://kinasephos.mbc.nctu.edu.tw/help.html#kinase) | - | E |
|  | 686 | NC | N | P | - | -4.4 | PKC, cdc2 | - | [ATM](http://kinasephos.mbc.nctu.edu.tw/help.html#kinase) | - | E |
|  | 691 | SC | N | P | - | - | - | - | - | - | E |
|  | 694 | SC | N | N | - | - | - | - | - | - | E |
|  | 708 | NC | N | N | - | - | - | - | - | - | E |
|  | 720 | C | N | P | - | - | - | - | - | - | E |
|  | 721 | NC | N | P | - | - | - | - | - | - | E |
|  | 736 | NC | N | Y | - | -4.9 | cdk5 | - | [INSR](http://kinasephos.mbc.nctu.edu.tw/help.html#kinase) | - | B |
|  | 741 | C | N | Y | - | 0.4 | - | - | [IKK](http://kinasephos.mbc.nctu.edu.tw/help.html#kinase) | - | E |
|  | 744 | C | N | Y | - | -1.5 | cdc2 | - | [CKI](http://kinasephos.mbc.nctu.edu.tw/help.html#kinase) | - | E |
|  | 745 | NC | N | Y | - | 4.6, -2.1 | GSK3, cdk5 | - | [CDK](http://kinasephos.mbc.nctu.edu.tw/help.html#kinase), [IKK](http://kinasephos.mbc.nctu.edu.tw/help.html#kinase) | - | E |
|  | 747 | SC | N | P | - | - | - | - | - | - | E |
|  | 763 | C | N | P | - | - | - | - | - | - | B |
|  | 766 | C | N | P | - | - | - | - | - | - | B |
|  | 770 | CS | N | Y | - | - | PKC | - | - | - | B |
|  | 792 | CS | N | Y | - | -5.5, -2.4 | PKA, cdc2 | - | [PKG](http://kinasephos.mbc.nctu.edu.tw/help.html#kinase), [CKI](http://kinasephos.mbc.nctu.edu.tw/help.html#kinase) | - | E |
|  | 794 | SC | Y | P | P | -3.3 | - | AMP_Kinase | [cdc2](http://kinasephos.mbc.nctu.edu.tw/help.html#kinase) | >1 | E |
|  | 795 | C | N | Y | - | - | - | - | - | - | E |
|  | 807 | C | N | Y | - | -1.4 | cdc2 | - | [IKK](http://kinasephos.mbc.nctu.edu.tw/help.html#kinase) | - | E |
|  | 808 | C | N | Y | - | 0.1 | CKI | - | [CKI](http://kinasephos.mbc.nctu.edu.tw/help.html#kinase) | - | E |
|  | 809 | C | N | Y | - | -0.7 | CKI | - | [CKI](http://kinasephos.mbc.nctu.edu.tw/help.html#kinase) | - | E |
|  | 810 | C | N | Y | - | - | - | - | - | - | E |
|  | 812 | C | N | Y | - | - | - | - | - | - | E |
|  | 813 | C | N | Y | - | -2.3 | - | - | [CKI](http://kinasephos.mbc.nctu.edu.tw/help.html#kinase) | - | E |
|  | 815 | CS | N | Y | - | - | CKI | - | - | - | E |
|  | 828 | NC | N | N | - | - | - | - | - | - | E |
|  | 853 | NC | N | N | - | - | - | - | - | - | B |
|  | 862 | C | N | Y | - | - | CKI, PKC, PKA, cdc2 | - | - | - | B |
|  | 869 | C | N | N | - | - | - | - | - | - | E |
|  | 892 | C | Y | Y | - | -3.3 | GSK3, cdk5 | - | [cdc2](http://kinasephos.mbc.nctu.edu.tw/help.html#kinase) | - | B |
|  | 903 | NC | N | N | - | - | cdc2 | - | - | - | E |
|  | 906 | NC | N | P | - | - | - | - | - | - | E |
|  | 910 | CS | N | N | - | -2.8 | cdc2 | - | [ATM](http://kinasephos.mbc.nctu.edu.tw/help.html#kinase) | - | E |
|  | 917 | NC | N | N | - | - | - | - | - | - | E |
|  | 918 | SC | N | N | - | - | p38MAPK, cdc2, GSK3, cdk5 | - | - | - | E |
|  | 920 | NC | N | Y | P | - | PKC | PKC alpha/beta/gamma | - | <1 | E |
|  | 925 | SC | N | Y | - | - | DNAPK, ATM | - | - | - | E |
|  | 957 | NC | N | N | - | - | - | - | - | - | E |
|  | 974 | CS | N | Y | - | - | cdc2 | - | - | - | E |
|  | 984 | NC | N | Y | - | - | PKC | - | - | - | E |
|  | 985 | SC | N | Y | - | - | CKI | - | - | - | E |
|  | 995 | SC | N | P | - | - | - | - | - | - | E |
|  | 1000 | SC | N | Y | - | - | - | - | - | - | E |
|  | 1005 | SC | N | Y | - | - | p38MAPK, cdk5 | - | - | - | E |
|  | 1011 | CS | N | Y | - | - | - | - | - | - | E |
|  | 1025 | SC | N | P | - | - | - | - | - | - | E |
|  | 1035 | SC | N | Y | - | - | cdc2 | - | - | - | E |
|  | 1036 | NC | N | Y | - | - | PKC, cdc2 | - | - | - | E |
|  | 1037 | CS | N | N | - | - | - | - | - | - | E |
|  | 1038 | C | N | P | - | - | - | - | - | - | E |
|  | 1041 | C | N | Y | P | -6.0 | - | PKC delta | [PKG](http://kinasephos.mbc.nctu.edu.tw/help.html#kinase) | <1 | E |
|  | 1043 | NC | N | Y | - | -1.2, 0.4 | p38MAPK, cdk5 | - | [cdc2](http://kinasephos.mbc.nctu.edu.tw/help.html#kinase), [IKK](http://kinasephos.mbc.nctu.edu.tw/help.html#kinase) | - | E |
|  | 1057 | NC | N | N | - | - | - | - | - | - | E |
|  | 1058 | NC | N | N | P | - | CKI | PKC mu | - | <1 | E |
|  | 1070 | C | N | N | - | - | cdc2 | - | - | - | E |
|  | 1078 | C | Y | Y | - | 0.3 | p38MAPK | - | [cdc2](http://kinasephos.mbc.nctu.edu.tw/help.html#kinase) | - | E |
|  | 1084 | C | N | Y | - | - | PKG | - | - | - | E |
|  | 1100 | C | N | Y | P | 6.5, 3.4 | RSK, PKA | Protein Kinase A | [PKA](http://kinasephos.mbc.nctu.edu.tw/help.html#kinase), [PKG](http://kinasephos.mbc.nctu.edu.tw/help.html#kinase) | >1 | E |
|  | 1101 | C | Y | Y | P | 4.4, -3.4, 3.8 | RSK, PKB | 14-3-3 Mode 1, Akt Kinase | [PKA](http://kinasephos.mbc.nctu.edu.tw/help.html#kinase), [PKG](http://kinasephos.mbc.nctu.edu.tw/help.html#kinase), [PKB](http://kinasephos.mbc.nctu.edu.tw/help.html#kinase) | >1 | E |
|  | 1105 | C | N | Y | - | - | - | - | - | - | E |
|  | 1106 | CS | N | P | - | - | cdc2 | - | - | - | E |
|  | 1109 | NC | N | P | - | -5.7 | CKI, PKC | - | [MAPK](http://kinasephos.mbc.nctu.edu.tw/help.html#kinase) | - | E |
|  | 1131 | SC | N | Y | - | - | - | - | - | - | E |
|  | 1132 | SC | N | Y | - | - | PKA, cdc2 | - | - | - | E |
|  | 1133 | NC | N | Y | - | 1.2 | CKII | - | [CKII](http://kinasephos.mbc.nctu.edu.tw/help.html#kinase) | - | E |
|  | 1134 | NC | N | Y | - | 3.6 | CKI, cdc2 | - | [Other_MDD](http://kinasephos.mbc.nctu.edu.tw/help.html#kinase) | - | E |
|  | 1135 | NC | N | Y | - | -3.9 | CKII, cdc2 | - | [ATM](http://kinasephos.mbc.nctu.edu.tw/help.html#kinase) | - | E |
|  | 1142 | C | N | Y | - | 3.9 | PKC, PKA | - | [PKA](http://kinasephos.mbc.nctu.edu.tw/help.html#kinase) | - | E |
|  | 1143 | C | N | P | P | 2.9 | RSK, PKA | 14-3-3 Mode 1 | [PKB](http://kinasephos.mbc.nctu.edu.tw/help.html#kinase) | <1 | B |
|  | 1145 | C | Y | Y | - | - | CKI | - | - | - | E |
|  | 1213 | NC | N | Y | - | -4.3 | - | - | [ATM](http://kinasephos.mbc.nctu.edu.tw/help.html#kinase) | - | E |
|  | 1216 | NC | N | N | - | - | - | - | - | - | E |
|  | 1217 | SC | N | Y | - | - | - | - | - | - | E |
|  | 1218 | SC | N | Y | - | - | PKC | - | - | - | E |
|  | 1222 | SC | Y | Y | - | - | PKA | - | - | - | E |
|  | 1223 | C | Y | Y | P | 2.2 | RSK, PKA | Protein Kinase A | [CaM-II](http://kinasephos.mbc.nctu.edu.tw/help.html#kinase) | >1 | B |
|  | 1227 | C | N | Y | - | - | - | - | - | - | E |
|  | 1231 | C | N | P | - | - | PKC | - | - | - | B |
|  | 1233 | NC | N | N | - | - | PKC | - | - | - | B |
| Threonine | 88 | C | N | P | - | - | CKII | - | - | - | B |
|  | 176 | NC | N | N | - | - | PKC | - | - | - | E |
|  | 188 | C | N | P | - | - | PKC | - | - | - | B |
|  | 191 | C | N | N | P | - | - | PKC delta, PKC epsilon | - | <1 | B |
|  | 231 | C | N | P | - | - | - | - | - | - | E |
|  | 252 | C | N | Y | - | - | CKII | - | - | - | B |
|  | 300 | SC | N | P | - | -2.6 | PKC | - | [PKC](http://kinasephos.mbc.nctu.edu.tw/help.html#kinase) | - | E |
|  | 305 | C | N | Y | P | -4.3 | PKA | 14-3-3 Mode 1 | [PKA](http://kinasephos.mbc.nctu.edu.tw/help.html#kinase) | >1 | E |
|  | 309 | C | N | N | - | - | - | - | - | - | E |
|  | 311 | C | N | N | - | - | PKC | - | - | - | E |
|  | 335 | C | N | Y | P | - | - | PKC delta | - | >1 | E |
|  | 351 | CS | N | P | - | - | - | - | - | - | E |
|  | 354 | CS | N | N | - | - | PKG | - | - | - | E |
|  | 387 | C | N | P | P | - | - | GSK3-improved, GSK3 Kinase | - | <1 | E |
|  | 397 | C | N | N | - | - | CKI | - | - | - | E |
|  | 403 | C | N | P | - | -3.4 | - | - | [PKA](http://kinasephos.mbc.nctu.edu.tw/help.html#kinase) | - | E |
|  | 446 | C | N | Y | - | -1.4, -5.4 | cdk5 | - | [PKA](http://kinasephos.mbc.nctu.edu.tw/help.html#kinase), [MAPK](http://kinasephos.mbc.nctu.edu.tw/help.html#kinase) | - | E |
|  | 453 | C | N | N | - | -2.6 | GSK3, cdk5 | - | [MAPK](http://kinasephos.mbc.nctu.edu.tw/help.html#kinase) | - | B |
|  | 475 | NC | N | P | - | - | - | - | - | - | E |
|  | 477 | NC | N | N | - | - | cdc2 | - | - | - | E |
|  | 495 | NC | N | Y | P | -2.8 | p38MAPK, cdk5 | Protein Kinase A | [PKA](http://kinasephos.mbc.nctu.edu.tw/help.html#kinase) | <1 | E |
|  | 498 | NC | N | P | - | - | PKC | - | - | - | E |
|  | 502 | NC | N | N | - | - | - | - | - | - | E |
|  | 525 | C | N | P | P | 1.4 | PKB, PKA, PKG | 14-3-3 Mode 1, Protein Kinase A | [PKA](http://kinasephos.mbc.nctu.edu.tw/help.html#kinase) | >1 | E |
|  | 530 | C | Y | N | - | - | PKC | - | - | - | E |
|  | 533 | C | N | N | - | - | - | - | - | - | E |
|  | 535 | CS | N | P | - | - | PKC | - | - | - | E |
|  | 539 | C | N | P | - | -1.5, 1.2 | GSK3, cdk5 | - | [MAPK](http://kinasephos.mbc.nctu.edu.tw/help.html#kinase), [CDK](http://kinasephos.mbc.nctu.edu.tw/help.html#kinase) | - | E |
|  | 552 | C | N | P | - | - | CKII | - | - | - | B |
|  | 579 | C | N | N | - | - | - | - | - | - | E |
|  | 605 | NC | N | N | - | - | - | - | - | - | E |
|  | 608 | C | N | N | - | - | CKII | - | - | - | E |
|  | 693 | CS | N | Y | - | - | PKC | - | - | - | E |
|  | 700 | C | N | N | - | - | - | - | - | - | E |
|  | 729 | CS | N | N | - | - | - | - | - | - | E |
|  | 743 | C | N | P | - | - | - | - | - | - | E |
|  | 774 | NC | N | Y | - | - | PKC | - | - | - | E |
|  | 793 | CS | N | P | - | - | - | - | - | - | E |
|  | 803 | NC | N | P | - | - | cdc2 | - | - | - | E |
|  | 811 | C | N | P | P | - | CKI, PKC | GSK3-improved | - | >1 | E |
|  | 847 | C | N | Y | - | - | - | - | - | - | E |
|  | 851 | NC | N | P | - | -2.8, -3.3 | - | - | [PKC](http://kinasephos.mbc.nctu.edu.tw/help.html#kinase), [MAPK](http://kinasephos.mbc.nctu.edu.tw/help.html#kinase) | - | E |
|  | 859 | C | N | P | - | - | PKG | - | - | - | B |
|  | 870 | C | N | P | P | -2.4 | PKC | 14-3-3 Mode 1 | [PKC](http://kinasephos.mbc.nctu.edu.tw/help.html#kinase) | >1 | E |
|  | 936 | NC | N | Y | P | - | CKII | Casein Kinase 2 | - | >1 | E |
|  | 938 | NC | N | N | - | - | CKII | - | - | - | E |
|  | 958 | NC | N | Y | - | - | CKII, cdc2 | - | - | - | E |
|  | 979 | SC | N | P | - | - | PKC | - | - | - | E |
|  | 991 | CS | N | N | - | -1.1 | - | - | [INSR](http://kinasephos.mbc.nctu.edu.tw/help.html#kinase) | - | E |
|  | 1004 | SC | N | Y | - | - | PKC | - | - | - | E |
|  | 1017 | CS | N | N | - | - | CKII | - | - | - | B |
|  | 1030 | SC | N | P | P | -4.8 | - | PKC epsilon | [PKA](http://kinasephos.mbc.nctu.edu.tw/help.html#kinase) | <1 | E |
|  | 1045 | NC | N | N | - | - | - | - | - | - | E |
|  | 1073 | C | N | N | - | - | cdc2 | - | - | - | B |
|  | 1103 | C | N | P | P | - | CKI, PKC | PKC delta | - | >1 | E |
|  | 1107 | SC | N | P | - | -1.6 | cdk5 | - | [MAPK](http://kinasephos.mbc.nctu.edu.tw/help.html#kinase) | - | E |
|  | 1111 | CS | Y | N | - | - | - | - | - | - | E |
|  | 1116 | NC | N | N | - | - | - | - | - | - | E |
|  | 1196 | NC | N | Y | - | 0.6, -1.5, 5.3 | p38MAPK | - | [cdc2](http://kinasephos.mbc.nctu.edu.tw/help.html#kinase), [MAPK](http://kinasephos.mbc.nctu.edu.tw/help.html#kinase), [Other_MDD](http://kinasephos.mbc.nctu.edu.tw/help.html#kinase) | - | E |
|  | 1219 | NC | N | Y | P | 0.8 | PKC, cdc2 | GSK3-improved | [PKC](http://kinasephos.mbc.nctu.edu.tw/help.html#kinase) | >1 | E |
| Tyrosine | 18 | C | N | Y | - | - | GSK3 | - | - | - | B |
|  | 46 | C | Y | P | - | - | - | - | - | - | B |
|  | 47 | C | N | Y | - | - | - | - | - | - | B |
|  | 87 | C | N | N | - | - | - | - | - | - | B |
|  | 107 | C | N | P | - | - | - | - | - | - | B |
|  | 151 | NC | N | Y | P | - | SRC | PDGFR Kin, PLCg N-terminal SH2, Fyn SH2, Src SH2, Lck SH2, Itk SH2 | - | <1 | B |
|  | 183 | C | N | N | - | - | - | - | - | - | B |
|  | 431 | C | N | Y | - | -4.5 | SRC | - | [INSR](http://kinasephos.mbc.nctu.edu.tw/help.html#kinase) | - | B |
|  | 465 | C | N | Y | P | 5.2 | INSR | PDGFR Kin, p85 SH2 | [INSR](http://kinasephos.mbc.nctu.edu.tw/help.html#kinase) | <1 | B |
|  | 483 | C | N | Y | - | - | - | - | - | - | E |
|  | 551 | C | N | Y | P | -3.2, -3.0 | - | Insulin Receptor Kinase, PDGFR Kin, Fgr Kinase, p85 SH2, Fyn SH2, Lck SH2 | [INSR](http://kinasephos.mbc.nctu.edu.tw/help.html#kinase), [Syk](http://kinasephos.mbc.nctu.edu.tw/help.html#kinase) | >1 | E |
|  | 558 | NC | N | P | - | - | - | - | - | - | E |
|  | 582 | C | N | P | - | - | - | - | - | - | E |
|  | 612 | C | Y | Y | P | 6.6, 1.5, 6.8 | SRC, INSR | PDGFR Kin, Insulin Receptor Kinase, p85 SH2 | [INSR](http://kinasephos.mbc.nctu.edu.tw/help.html#kinase), [Syk](http://kinasephos.mbc.nctu.edu.tw/help.html#kinase), [Other_MDD](http://kinasephos.mbc.nctu.edu.tw/help.html#kinase) | <1 | E |
|  | 632 | C | Y | Y | P | 8.0, -1.2, 1.7 | INSR | PDGFR Kin, Insulin Receptor Kinase, p85 SH2 | [INSR](http://kinasephos.mbc.nctu.edu.tw/help.html#kinase), [Syk](http://kinasephos.mbc.nctu.edu.tw/help.html#kinase), [Jak](http://kinasephos.mbc.nctu.edu.tw/help.html#kinase) | <1 | B |
|  | 662 | C | Y | Y | P | 3.8, 3.1 | INSR | Insulin Receptor Kinase, PDGFR Kin, p85 SH2 | [INSR](http://kinasephos.mbc.nctu.edu.tw/help.html#kinase), [Syk](http://kinasephos.mbc.nctu.edu.tw/help.html#kinase) | <1 | B |
|  | 695 | C | N | Y | P | - | - | SHIP SH2 | - | >1 | E |
|  | 732 | C | N | Y | P | 2.6 | EGFR, INSR | Insulin Receptor Kinase, p85 SH2, Grb2 SH2 | [INSR](http://kinasephos.mbc.nctu.edu.tw/help.html#kinase) | <1 | E |
|  | 750 | C | N | P | P | -1.1 | - | PDGFR Kin, Fgr Kinase, Itk SH2, Abl SH2 | [Syk](http://kinasephos.mbc.nctu.edu.tw/help.html#kinase) | <1 | E |
|  | 751 | NC | N | P | - | -1.4 | SRC, INSR | - | [Syk](http://kinasephos.mbc.nctu.edu.tw/help.html#kinase) | - | E |
|  | 764 | C | N | P | - | - | - | - | - | - | B |
|  | 765 | CS | N | N | - | - | - | - | - | - | E |
|  | 800 | C | N | P | - | - | - | - | - | - | E |
|  | 820 | NC | N | N | - | - | - | - | - | - | E |
|  | 896 | C | Y | Y | P | -0.5, 2.6, 5.1 | - | PDGFR Kin, Fgr Kinase, Grb2 SH2, PLCg N-terminal SH2 | [Src](http://kinasephos.mbc.nctu.edu.tw/help.html#kinase), [INSR](http://kinasephos.mbc.nctu.edu.tw/help.html#kinase), [Other_MDD](http://kinasephos.mbc.nctu.edu.tw/help.html#kinase) | <1 | E |
|  | 908 | NC | N | Y | - | 0.8 | - | - | [Jak](http://kinasephos.mbc.nctu.edu.tw/help.html#kinase) | - | E |
|  | 941 | C | N | Y | P | 0.9, 4.4, 1.7, 0.2 | - | PDGFR Kin, Insulin Receptor Kinase, Fgr Kinase, p85 SH2 | [Src](http://kinasephos.mbc.nctu.edu.tw/help.html#kinase), [INSR](http://kinasephos.mbc.nctu.edu.tw/help.html#kinase), [Syk](http://kinasephos.mbc.nctu.edu.tw/help.html#kinase), [Jak](http://kinasephos.mbc.nctu.edu.tw/help.html#kinase) | >1 | B |
|  | 989 | C | N | Y | P | 6.4, -2.7 | - | Insulin Receptor Kinase, p85 SH2 | [INSR](http://kinasephos.mbc.nctu.edu.tw/help.html#kinase), [Syk](http://kinasephos.mbc.nctu.edu.tw/help.html#kinase) | <1 | E |
|  | 1001 | C | N | Y | - | - | - | - | - | - | E |
|  | 1012 | NC | N | Y | - | - | - | - | - | - | B |
|  | 1179 | C | N | Y | P | 0.1 | - | PLCg N-terminal SH2 | [INSR](http://kinasephos.mbc.nctu.edu.tw/help.html#kinase) | <1 | B |
|  | 1229 | C | N | Y | - | 0.3 | INSR | - | [INSR](http://kinasephos.mbc.nctu.edu.tw/help.html#kinase) | - | B |

| Table S2  **Experimental and predicted phosphorylation, kinases involved and surface accessibility for Ser/ Thr/ Tyr residues of human IRS-2. Abbreviation used in table: PS, position; EV, experimentally verified; C, conserved; CS, conserved substitutions; SC, semi-conserved; NC, non-conserved; Y, yes (threshold > 0.5) (predicted or experimentally verified phosphorylated residues); P, probable (threshold = 0.1~0.5) (may be phosphorylated residues); N, No (threshold < 0.1) (not predicted or not experimentally verified phosphorylated residues); E, exposed surface; B, buried surface; >1, high surface accessibility; <1, low surface accessibility, 1 = low potential for solvent accessibility, 2 = high potential for solvent accessibility.** | | | | | | | | | | | |
| --- | --- | --- | --- | --- | --- | --- | --- | --- | --- | --- | --- |
| Substrate | | | Phosphorylation | | | | Kinases Involved | | | Surface Accessibility | |
| Residue | PS | Cons. | EV | Netphos | Scansite | Kinasephos  (score) | NetphosK | Scansite | Kinasephos | Scansite | NetSurfP |
|  | 3 | NC | N | N | - | - | p38MAPK, cdk5 | -­ | - | - | E |
| Serine | 14 | NC | N | P | - | -2.3 | - | - | [IKK](http://kinasephos.mbc.nctu.edu.tw/help.html#kinase) | - | E |
|  | 30 | NC | N | P | - | - | PKC | - | - | - | E |
|  | 66 | NC | N | P | - | - | - | - | - | - | E |
|  | 78 | NC | N | Y | - | - | PKC | - | - | - | B |
|  | 84 | NC | N | Y | - | - | PKC | - | - | - | E |
|  | 144 | NC | N | Y | - | - | - | - | - | - | E |
|  | 162 | NC | N | P | - | - | - | - | - | - | E |
|  | 164 | NC | N | P | - | - | - | - | - | - | E |
|  | 166 | NC | N | N | - | - | - | - | - | - | E |
|  | 174 | NC | N | N | - | - | cdc2 | - | - | - | B |
|  | 183 | NC | N | N | - | - | - | - | - | - | B |
|  | 210 | NC | N | N | - | - | PKA, cdc2 | - | - | - | E |
|  | 222 | NC | N | N | - | - | PKC | - | - | - | B |
|  | 237 | NC | N | N | - | - | - | - | - | - | B |
|  | 251 | NC | N | N | - | - | PKA | - | - | - | B |
|  | 253 | NC | N | Y | P | - | cdc2 | PKC delta | - | <1 | B |
|  | 262 | NC | N | N | - | - | - | - | - | - | E |
|  | 277 | NC | N | Y | - | - | - | - | - | - | E |
|  | 304 | NC | Y | Y | - | -0.7 | PKA | - | [IKK](http://kinasephos.mbc.nctu.edu.tw/help.html#kinase) | - | E |
|  | 306 | NC | Y | Y | P | -3.8, -4.5, 1.2 | RSK,DNAPK, PKB | 14-3-3 Mode 1 | [PKG](http://kinasephos.mbc.nctu.edu.tw/help.html#kinase), [ATM](http://kinasephos.mbc.nctu.edu.tw/help.html#kinase), [IKK](http://kinasephos.mbc.nctu.edu.tw/help.html#kinase) | >1 | E |
|  | 308 | NC | Y | Y | - | - | PKC | - | - | - | E |
|  | 309 | NC | N | Y | - | 0.4 | cdc2 | - | [CKII](http://kinasephos.mbc.nctu.edu.tw/help.html#kinase) | - | E |
|  | 311 | NC | N | Y | - | -2.2 | cdc2 | - | [CKI](http://kinasephos.mbc.nctu.edu.tw/help.html#kinase) | - | E |
|  | 312 | NC | Y | P | - | - | - | - | - | - | E |
|  | 318 | NC | N | Y | - | - | - | - | - | - | E |
|  | 334 | NC | Y | Y | - | - | DNAPK | - | - | - | E |
|  | 342 | NC | Y | Y | - | 3.0, -4.7 | PKA, PKG, cdc2 | - | [PKA](http://kinasephos.mbc.nctu.edu.tw/help.html#kinase), [PKG](http://kinasephos.mbc.nctu.edu.tw/help.html#kinase) | - | E |
|  | 346 | NC | N | Y | P | 0.1, 2.4 | RSK, PKB, PKC, PKA | Akt Kinase, PKC epsilon | [CaM-II](http://kinasephos.mbc.nctu.edu.tw/help.html#kinase), [IKK](http://kinasephos.mbc.nctu.edu.tw/help.html#kinase) | <1 | E |
|  | 357 | NC | N | N | - | - | PKG | - | - | - | E |
|  | 358 | NC | N | N | - | - | PKC | - | - | - | E |
|  | 365 | NC | Y | Y | P | - | RSK, PKB | 14-3-3 Mode 1, Akt Kinase | - | >1 | E |
|  | 384 | NC | Y | Y | - | 1.0 | PKA | - | [IKK](http://kinasephos.mbc.nctu.edu.tw/help.html#kinase) | - | E |
|  | 388 | NC | Y | P | - | 2.2, 2.8, 0.4 | p38MAPK, GSK3, cdk5 | - | [cdc2](http://kinasephos.mbc.nctu.edu.tw/help.html#kinase), [CDK](http://kinasephos.mbc.nctu.edu.tw/help.html#kinase), [IKK](http://kinasephos.mbc.nctu.edu.tw/help.html#kinase) | - | E |
|  | 391 | NC | Y | Y | P | -1.4, 3.7, -4.2, -6.6 | p38MAPK, GSK3, cdk5 | - | [cdc2](http://kinasephos.mbc.nctu.edu.tw/help.html#kinase), [MAPK](http://kinasephos.mbc.nctu.edu.tw/help.html#kinase), [ATM](http://kinasephos.mbc.nctu.edu.tw/help.html#kinase), [MAPK](http://kinasephos.mbc.nctu.edu.tw/help.html#kinase) | - | E |
|  | 400 | NC | N | Y | - | - | - | - | - | - | E |
|  | 402 | NC | N | P | - | - | - | - | - | - | E |
|  | 406 | NC | N | Y | - | - | - | - | - | - | E |
|  | 414 | NC | N | P | - | - | PKA | - | - | - | E |
|  | 428 | NC | N | Y | - | - | - | - | - | - | B |
|  | 430 | NC | N | Y | - | - | cdc2 | - | - | - | E |
|  | 432 | NC | N | Y | P | 2.5 | DNAPK | 14-3-3 Mode 1 | [PKB](http://kinasephos.mbc.nctu.edu.tw/help.html#kinase) | <1 | E |
|  | 438 | NC | N | Y | - | - | GSK3, cdk5 | - | - | - | E |
|  | 444 | NC | N | Y | - | 1.6, -6.5 | GSK3, cdk5 | - | [MAPK](http://kinasephos.mbc.nctu.edu.tw/help.html#kinase) | - | E |
|  | 447 | NC | N | Y | - | -0.3 | CKI,DNAPK, PKC | - | [IKK](http://kinasephos.mbc.nctu.edu.tw/help.html#kinase) | - | E |
|  | 449 | NC | N | Y | - | - | cdc2 | - | - | - | E |
|  | 450 | NC | N | P | - | - | CKI, PKC | - | - | - | E |
|  | 451 | NC | N | Y | - | -2.2, -3.9 | cdc2 | - | [CKI](http://kinasephos.mbc.nctu.edu.tw/help.html#kinase), [ATM](http://kinasephos.mbc.nctu.edu.tw/help.html#kinase) | - | E |
|  | 452 | NC | N | Y | - | 0.4, -1.4 | cdc2 | - | [CKII](http://kinasephos.mbc.nctu.edu.tw/help.html#kinase), [CKI](http://kinasephos.mbc.nctu.edu.tw/help.html#kinase) | - | E |
|  | 456 | NC | N | P | - | -4.3 | - | - | [ATM](http://kinasephos.mbc.nctu.edu.tw/help.html#kinase) | - | E |
|  | 458 | NC | N | P | - | - | DNAPK,PKC, cdc2 | - | - | - | E |
|  | 482 | NC | N | Y | - | - | PKA, cdc2 | - | - | - | E |
|  | 483 | NC | N | Y | - | -0.0, -2.7, -1.6 | - | - | [CKII](http://kinasephos.mbc.nctu.edu.tw/help.html#kinase), [ATM](http://kinasephos.mbc.nctu.edu.tw/help.html#kinase), [IKK](http://kinasephos.mbc.nctu.edu.tw/help.html#kinase) | - | E |
|  | 485 | NC | N | Y | - | -1.0 | PKC | - | [IKK](http://kinasephos.mbc.nctu.edu.tw/help.html#kinase) | - | E |
|  | 487 | NC | N | Y | P | -2.3 | - | GSK3-improved | [IKK](http://kinasephos.mbc.nctu.edu.tw/help.html#kinase) | <1 | E |
|  | 489 | NC | N | Y | - | -3.5, -1.8 | - | - | [ATM](http://kinasephos.mbc.nctu.edu.tw/help.html#kinase), [IKK](http://kinasephos.mbc.nctu.edu.tw/help.html#kinase) | - | E |
|  | 491 | NC | N | Y | - | -2.0, -2.1, 1.5 | cdc2, GSK3, cdk5 | - | [cdc2](http://kinasephos.mbc.nctu.edu.tw/help.html#kinase), [ATM](http://kinasephos.mbc.nctu.edu.tw/help.html#kinase), [IKK](http://kinasephos.mbc.nctu.edu.tw/help.html#kinase) | - | E |
|  | 493 | NC | N | P | - | -2.1 | - | - | [ATM](http://kinasephos.mbc.nctu.edu.tw/help.html#kinase) | - | E |
|  | 499 | NC | N | P | - | - | CKII | - | - | - | E |
|  | 505 | NC | N | N | - | - | - | - | - | - | E |
|  | 506 | NC | N | Y | - | -2.5 | GSK3, cdk5 | - | [ATM](http://kinasephos.mbc.nctu.edu.tw/help.html#kinase) | - | E |
|  | 515 | NC | N | N | - | - | PKC | - | - | - | E |
|  | 518 | NC | Y | N | - | - | - | - | - | - | E |
|  | 523 | NC | Y | Y | - | - | - | - | - | - | E |
|  | 550 | NC | Y | Y | - | - | RSK | - | - | - | E |
|  | 555 | NC | N | Y | - | - | PKC | - | - | - | E |
|  | 560 | NC | Y | Y | P | -2.7 | RSK, PKA | Protein Kinase A | [PKG](http://kinasephos.mbc.nctu.edu.tw/help.html#kinase) | <1 | E |
|  | 577 | NC | Y | Y | P | 0.1 | RSK, PKB, PKA | 14-3-3 Mode 1, Akt Kinase | [IKK](http://kinasephos.mbc.nctu.edu.tw/help.html#kinase) | >1 | E |
|  | 591 | NC | N | P | - | - | - | - | - | - | E |
|  | 592 | NC | N | N | - | -2.5 | cdc2 | - | [IKK](http://kinasephos.mbc.nctu.edu.tw/help.html#kinase) | - | E |
|  | 594 | NC | Y | Y | - | - | CKII | - | - | - | E |
|  | 606 | NC | N | Y | - | - | PKC | - | - | - | E |
|  | 608 | NC | Y | Y | - | - | cdc2 | - | - | - | E |
|  | 615 | NC | N | N | - | -0.8 | PKC | - | [IKK](http://kinasephos.mbc.nctu.edu.tw/help.html#kinase) | - | E |
|  | 619 | NC | N | N | - | -3.0 | - | - | [cdc2](http://kinasephos.mbc.nctu.edu.tw/help.html#kinase) | - | E |
|  | 620 | NC | Y | Y | - | - | cdk5 | - | - | - | E |
|  | 639 | NC | N | Y | - | - | PKC | - | - | - | E |
|  | 642 | NC | N | Y | - | - | - | - | - | - | E |
|  | 643 | NC | N | Y | - | -1.7 | RSK, PKA | - | [CKI](http://kinasephos.mbc.nctu.edu.tw/help.html#kinase) | - | E |
|  | 644 | NC | N | Y | - | -1.6 | RSK, PKA | - | [CKI](http://kinasephos.mbc.nctu.edu.tw/help.html#kinase) | - | E |
|  | 645 | NC | N | P |  | - | cdc2 | - | - | - | E |
|  | 665 | NC | N | Y | - | - | cdc2 | - | - | - | E |
|  | 667 | NC | N | N | - | - | - | - | - | - | E |
|  | 669 | NC | N | Y | - | - | - | - | - | - | E |
|  | 672 | NC | N | N | - | - | - | - | - | - | E |
|  | 679 | NC | Y | P | - | - | p38MAPK, GSK3, cdk5 | - | - | - | E |
|  | 682 | NC | N | Y | - | 0.2 | PKC | - | [IKK](http://kinasephos.mbc.nctu.edu.tw/help.html#kinase) | - | E |
|  | 684 | NC | N | P | - | - | - | - | - | - | E |
|  | 704 | NC | N | N | - | - | PKG | - | - | - | E |
|  | 714 | NC | Y | N | - | - | - | - | - | - | E |
|  | 723 | NC | N | Y | - | - | PKC | - | - | - | E |
|  | 730 | NC | Y | N | - | - | PKC | - | - | - | E |
|  | 731 | NC | Y | Y | - | 0.1 | GSK3 | - | [cdc2](http://kinasephos.mbc.nctu.edu.tw/help.html#kinase) | - | E |
|  | 735 | NC | Y | Y | - | 4.3, -2.0 | CKII | - | [CKII](http://kinasephos.mbc.nctu.edu.tw/help.html#kinase), [CKI](http://kinasephos.mbc.nctu.edu.tw/help.html#kinase) | - | E |
|  | 736 | NC | Y | Y | - | -1.5 | cdk5 | - | [IKK](http://kinasephos.mbc.nctu.edu.tw/help.html#kinase) | - | E |
|  | 740 | NC | N | Y | - | - | - | - | - | - | E |
|  | 749 | NC | N | N | - | - | PKC | - | - | - | E |
|  | 752 | NC | N | Y | - | - | cdc2 | - | - | - | E |
|  | 770 | NC | Y | Y | - | - | cdk5 | - | - | - | B |
|  | 772 | NC | Y | P | - | - | - | - | - | - | E |
|  | 785 | NC | N | N | - | - | - | - | - | - | E |
|  | 804 | NC | N | N | - | - | cdc2 | - | - | - | E |
|  | 805 | NC | N | N | - | - | - | - | - | - | E |
|  | 809 | NC | N | Y | - | - | PKC | - | - | - | E |
|  | 820 | NC | N | N | - | - | PKA | - | - | - | E |
|  | 827 | NC | N | N | - | - | - | - | - | - | E |
|  | 828 | NC | Y | P | - | -0.4 | - | - | [cdc2](http://kinasephos.mbc.nctu.edu.tw/help.html#kinase) | - | E |
|  | 848 | NC | N | P | - | 0.1 | DNAPK, ATM | - | [ATM](http://kinasephos.mbc.nctu.edu.tw/help.html#kinase) | - | E |
|  | 852 | NC | N | P | - | - | - | - | - | - | E |
|  | 868 | NC | N | P | - | 4.2, 2.0 | cdc2, GSK3, cdk5 | - | [cdc2](http://kinasephos.mbc.nctu.edu.tw/help.html#kinase), [CDK](http://kinasephos.mbc.nctu.edu.tw/help.html#kinase) | - | E |
|  | 873 | C | N | Y | - | - | PKA | - | - | - | E |
|  | 894 | C | Y | Y | - | -5.0 | RSK, CKI, PKA | - | [PKG](http://kinasephos.mbc.nctu.edu.tw/help.html#kinase) | - | E |
|  | 900 | CS | N | P | - | - | cdc2 | - | - | - | E |
|  | 903 | C | N | Y | - | - | - | - | - | - | E |
|  | 915 | C | Y | Y | - | -3.3 | GSK3, cdk5 | - | [cdc2](http://kinasephos.mbc.nctu.edu.tw/help.html#kinase) | - | E |
|  | 932 | C | N | Y | - | 0.1, -3.1 | p38MAPK, PKA, GSK3, cdk5 | - | [MAPK](http://kinasephos.mbc.nctu.edu.tw/help.html#kinase), [ATM](http://kinasephos.mbc.nctu.edu.tw/help.html#kinase) | - | E |
|  | 941 | C | N | N | - | - | - | - | - | - | E |
|  | 944 | C | N | P | - | - | - | - | - | - | E |
|  | 945 | C | N | P | - | - | cdc2 | - | - | - | E |
|  | 946 | C | N | N | - | - | cdc2 | - | - | - | E |
|  | 947 | C | N | P | - | -0.7 | cdc2 | - | [CKI](http://kinasephos.mbc.nctu.edu.tw/help.html#kinase) | - | E |
|  | 950 | C | N | Y | - | - | - | - | - | - | E |
|  | 952 | C | N | P | - | - | - | - | - | - | E |
|  | 953 | C | N | Y | P | -0.4, -4.1 | CKI, p38MAPK, cdc2, GSK3, cdk5 | GSK3-improved | [cdc2](http://kinasephos.mbc.nctu.edu.tw/help.html#kinase), [ATM](http://kinasephos.mbc.nctu.edu.tw/help.html#kinase) | <1 | E |
|  | 956 | C | N | Y | - | -1.6 | CKI, PKC | - | [IKK](http://kinasephos.mbc.nctu.edu.tw/help.html#kinase) | - | E |
|  | 957 | C | N | Y | P | - | CKI | PKC delta | - | <1 | E |
|  | 960 | C | N | Y | - | - | PKC | - | - | - | E |
|  | 966 | C | N | N | - | - | cdc2 | - | - | - | E |
|  | 967 | C | N | Y | - | - | - | - | - | - | E |
|  | 969 | C | N | Y | - | - | cdc2 | - | - | - | E |
|  | 973 | C | Y | Y | - | 0.1, -5.8, 0.5 | GSK3, cdk5 | - | [CaM-II](http://kinasephos.mbc.nctu.edu.tw/help.html#kinase), [PKG](http://kinasephos.mbc.nctu.edu.tw/help.html#kinase), [IKK](http://kinasephos.mbc.nctu.edu.tw/help.html#kinase) | - | E |
|  | 976 | C | N | Y | - | - | cdc2 | - | - | - | E |
|  | 984 | C | N | Y | - | - | PKC | - | - | - | E |
|  | 985 | C | N | Y | - | -3.0 | - | - | [cdc2](http://kinasephos.mbc.nctu.edu.tw/help.html#kinase) | - | E |
|  | 988 | C | N | Y | P | -1.8, -3.8 | p38MAPK, GSK3, cdk5 | - | [cdc2](http://kinasephos.mbc.nctu.edu.tw/help.html#kinase), [ATM](http://kinasephos.mbc.nctu.edu.tw/help.html#kinase) | - | E |
|  | 995 | C | N | P | - | - | - | - | - | - | E |
|  | 1001 | C | N | N | - | - | CKI, DNAPK | - | - | - | E |
|  | 1007 | C | N | Y | - | -3.3, 3.3, -4.4, -6.1 | p38MAPK, GSK3 | - | [cdc2](http://kinasephos.mbc.nctu.edu.tw/help.html#kinase), [MAPK](http://kinasephos.mbc.nctu.edu.tw/help.html#kinase), [ATM](http://kinasephos.mbc.nctu.edu.tw/help.html#kinase), [MAPK](http://kinasephos.mbc.nctu.edu.tw/help.html#kinase) | - | E |
|  | 1011 | C | N | N | - | - | - | - | - | - | E |
|  | 1012 | C | N | Y | - | 1.7 | cdc2, cdk5 | - | [cdc2](http://kinasephos.mbc.nctu.edu.tw/help.html#kinase) | - | E |
|  | 1022 | C | N | Y | - | - | PKG | - | - | - | E |
|  | 1024 | C | N | Y | P | -1.1, 3.0, -2.9 | p38MAPK, GSK3, cdk5 | GSK3-improved, GSK3 Kinase | [cdc2](http://kinasephos.mbc.nctu.edu.tw/help.html#kinase), [CDK](http://kinasephos.mbc.nctu.edu.tw/help.html#kinase), [ATM](http://kinasephos.mbc.nctu.edu.tw/help.html#kinase) | >1 | E |
|  | 1026 | C | N | Y | - | - | - | - | - | - | E |
|  | 1027 | C | N | Y | - | - | - | - | - | - | E |
|  | 1028 | NC | N | P | - | - | - | - | - | - | E |
|  | 1048 | CS | N | Y | - | - | - | - | - | - | E |
|  | 1060 | C | N | Y | - | - | - | - | - | - | E |
|  | 1061 | C | N | P | - | - | - | - | - | - | E |
|  | 1063 | C | N | Y | - | -0.4 | CKI | - | [CKI](http://kinasephos.mbc.nctu.edu.tw/help.html#kinase) | - | E |
|  | 1064 | C | N | P | - | -0.9 | CKI | - | [CKI](http://kinasephos.mbc.nctu.edu.tw/help.html#kinase) | - | E |
|  | 1100 | C | Y | Y | - | -3.5, 0.7 | RSK, p38MAPK, PKA, cdk5 | - | [cdc2](http://kinasephos.mbc.nctu.edu.tw/help.html#kinase), [PKB](http://kinasephos.mbc.nctu.edu.tw/help.html#kinase) | - | E |
|  | 1103 | C | Y | P | - | - | - | - | - | - | E |
|  | 1109 | C | Y | Y | P | - | PKA | Protein Kinase A, PKC epsilon | - | <1 | E |
|  | 1115 | C | N | Y | - | - | CKII | - | - | - | E |
|  | 1124 | C | N | N | - | -0.0 | DNAPK, ATM | - | [ATM](http://kinasephos.mbc.nctu.edu.tw/help.html#kinase) | - | E |
|  | 1148 | C | Y | Y | P | 6.5, 3.4 | RSK,PKA | Protein Kinase A | [PKA](http://kinasephos.mbc.nctu.edu.tw/help.html#kinase), [PKG](http://kinasephos.mbc.nctu.edu.tw/help.html#kinase) | >1 | E |
|  | 1149 | C | Y | Y | P | 4.4, -3.4, 3.8 | RSK, PKB, PKA | 14-3-3 Mode 1 | [PKA](http://kinasephos.mbc.nctu.edu.tw/help.html#kinase), [PKG](http://kinasephos.mbc.nctu.edu.tw/help.html#kinase), [PKB](http://kinasephos.mbc.nctu.edu.tw/help.html#kinase) | >1 | E |
|  | 1153 | C | N | Y | - | -0.8 | - | - | [CKI](http://kinasephos.mbc.nctu.edu.tw/help.html#kinase) | - | E |
|  | 1154 | C | N | N | - | - | cdc2 | - | - | - | E |
|  | 1162 | C | Y | Y | P | -0.4 | CKI, GSK3, cdk5 | - | [cdc2](http://kinasephos.mbc.nctu.edu.tw/help.html#kinase) | - | E |
|  | 1164 | C | Y | P | - | - | PKC | - | - | - | E |
|  | 1174 | C | Y | P | P | 0.5, 0.9 | PKA, PKG | 14-3-3 Mode 1 | [PKG](http://kinasephos.mbc.nctu.edu.tw/help.html#kinase), [PKB](http://kinasephos.mbc.nctu.edu.tw/help.html#kinase) | <1 | E |
|  | 1176 | C | Y | Y | - | - | - | - | - | - | E |
|  | 1181 | C | N | Y | - | - | PKC | - | - | - | E |
|  | 1185 | C | Y | Y | - | -5.2, -2.5 | - | - | [PKG](http://kinasephos.mbc.nctu.edu.tw/help.html#kinase), [CKI](http://kinasephos.mbc.nctu.edu.tw/help.html#kinase) | - | E |
|  | 1186 | C | N | Y | P | -5.4 | PKA | 14-3-3 Mode 1 | [PKG](http://kinasephos.mbc.nctu.edu.tw/help.html#kinase) | >1 | E |
|  | 1203 | C | Y | Y | - | -2.1, 1.6, 4.6 | p38MAPK, GSK3, cdk5 | - | [cdc2](http://kinasephos.mbc.nctu.edu.tw/help.html#kinase), [MAPK](http://kinasephos.mbc.nctu.edu.tw/help.html#kinase), [Other_MDD](http://kinasephos.mbc.nctu.edu.tw/help.html#kinase) | - | E |
|  | 1234 | SC | N | N | - | - | - | - | - | - | E |
|  | 1237 | C | N | Y | P | - | p38MAPK, cdk5 | - | - | - | E |
|  | 1244 | C | N | Y | P | 3.6, -4.5 | RSK, PKA | 14-3-3 Mode 1 | [PKA](http://kinasephos.mbc.nctu.edu.tw/help.html#kinase) | <1 | E |
|  | 1282 | SC | N | N | - | - | - | - | - | - | E |
|  | 1283 | C | Y | Y | - | - | PKC | - | - | - | E |
|  | 1289 | C | N | N | P | 1.6 | CKI, PKA | 14-3-3 Mode 1 | [CaM-II](http://kinasephos.mbc.nctu.edu.tw/help.html#kinase) | <1 | E |
|  | 1295 | SC | N | N | - | - | - | - | - | - | E |
|  | 1301 | SC | N | P | - | - | CKI | - | - | - | E |
|  | 1322 | C | N | P | - | - | - | - | - | - | B |
|  | 1327 | C | N | N | - | - | - | - | - | - | B |
| Threonine | 61 | NC | N | N | - | - | CKI | - | - | - | E |
|  | 117 | NC | N | Y | - | - | CKII, PKC, PKG | - | - | - | B |
|  | 140 | NC | N | Y | - | - | - | - | - | - | B |
|  | 191 | NC | N | N | - | - | - | - | - | - | B |
|  | 214 | NC | N | N | - | - | - | - | - | - | E |
|  | 225 | NC | N | N | - | - | - | - | - | - | B |
|  | 239 | NC | N | N | - | - | - | - | - | - | E |
|  | 265 | NC | N | P | - | - | - | - | - | - | B |
|  | 286 | NC | N | Y | - | - | CKII | - | - | - | E |
|  | 314 | NC | N | N | - | - | - | - | - | - | B |
|  | 336 | NC | N | P | - | - | - | - | - | - | E |
|  | 344 | NC | N | P | P | -1.6 | PKA, PKG | 14-3-3 Mode 1 | [PKA](http://kinasephos.mbc.nctu.edu.tw/help.html#kinase) | >1 | B |
|  | 350 | NC | Y | P | - | -2.4 | cdc2, GSK3, cdk5 | - | [MAPK](http://kinasephos.mbc.nctu.edu.tw/help.html#kinase) | - | E |
|  | 363 | NC | Y | Y | P | - | PKG | 14-3-3 Mode 1 | - | >1 | B |
|  | 404 | NC | N | P | P | - | - | 14-3-3 Mode 1, PKC mu | - | <1 | E |
|  | 443 | NC | N | Y | P | - | - | GSK3-improved | - | <1 | E |
|  | 520 | NC | Y | Y | - | -3.4 | - | - | [MAPK](http://kinasephos.mbc.nctu.edu.tw/help.html#kinase) | - | E |
|  | 527 | NC | Y | P | - | -1.2 | GSK3, cdk5 | - | [MAPK](http://kinasephos.mbc.nctu.edu.tw/help.html#kinase) | - | E |
|  | 544 | NC | N | Y | - | -3.4 | - | - | [INSR](http://kinasephos.mbc.nctu.edu.tw/help.html#kinase) | - | E |
|  | 575 | NC | N | P | P | -3.0 | PKA, PKG, cdc2 | 14-3-3 Mode 1, Protein Kinase A | [PKA](http://kinasephos.mbc.nctu.edu.tw/help.html#kinase) | >1 | E |
|  | 579 | NC | Y | N | - | - | PKC | - | - | - | E |
|  | 580 | NC | Y | Y | P | - | p38MAPK, cdk5 | - | - | - | E |
|  | 599 | NC | N | P | P | - | cdc2 | PKC delta | - | >1 | E |
|  | 604 | NC | N | P | P | - | - | PKC epsilon | - | <1 | E |
|  | 657 | NC | N | Y | - | -5.5 | p38MAPK, GSK3, cdk5 | - | [MAPK](http://kinasephos.mbc.nctu.edu.tw/help.html#kinase) | - | E |
|  | 713 | NC | Y | P | - | - | - | - | - | - | E |
|  | 719 | NC | N | N | - | - | PKC | - | - | - | E |
|  | 776 | NC | N | N | - | - | - | - | - | - | E |
|  | 777 | NC | Y | Y | - | - | - | - | - | - | E |
|  | 779 | NC | Y | Y | - | -0.1 | p38MAPK, cdk5 | - | [MAPK](http://kinasephos.mbc.nctu.edu.tw/help.html#kinase) | - | E |
|  | 815 | NC | N | P | - | - | - | - | - | - | E |
|  | 844 | NC | N | Y | - | 0.1 | p38MAPK, GSK3 | - | [MAPK](http://kinasephos.mbc.nctu.edu.tw/help.html#kinase) | - | E |
|  | 859 | NC | N | N | - | - | PKC | - | - | - | E |
|  | 891 | C | N | N | - | - | PKA | - | - | - | E |
|  | 962 | C | N | P | - | -3.4 | p38MAPK, cdk5 | - | [MAPK](http://kinasephos.mbc.nctu.edu.tw/help.html#kinase) | - | E |
|  | 965 | C | N | Y | - | - | - | - | - | - | E |
|  | 1052 | C | N | P | - | - | - | - | - | - | E |
|  | 1066 | SC | N | N | - | - | CKII | - | - | - | E |
|  | 1073 | C | N | P | - | -2.1 | - | - | [INSR](http://kinasephos.mbc.nctu.edu.tw/help.html#kinase) | - | E |
|  | 1082 | SC | N | P | - | -0.8, -2.8 | p38MAPK, cdc2, cdk5 | - | [cdc2](http://kinasephos.mbc.nctu.edu.tw/help.html#kinase), [MAPK](http://kinasephos.mbc.nctu.edu.tw/help.html#kinase) | - | E |
|  | 1102 | C | N | P | - | - | PKC | - | - | - | E |
|  | 1151 | C | Y | P | P | - | CKI, PKC, cdc2 | PKC delta | - | >1 | E |
|  | 1155 | C | N | P | - | - | PKC, cdc2 | - | - | - | E |
|  | 1156 | C | N | P | - | - | - | - | - | - | E |
|  | 1157 | C | N | Y | P | - | PKC | PKC epsilon | - | >1 | E |
|  | 1159 | C | Y | Y | - | -3.9, 2.1 | cdk5 | - | [MAPK](http://kinasephos.mbc.nctu.edu.tw/help.html#kinase), [CDK](http://kinasephos.mbc.nctu.edu.tw/help.html#kinase) | - | E |
|  | 1202 | C | Y | P | - | - | - | - | - | - | E |
|  | 1221 | SC | N | Y | P | -5.8 | p38MAPK, GSK3, cdk5 | - | [MAPK](http://kinasephos.mbc.nctu.edu.tw/help.html#kinase) | - | E |
|  | 1243 | C | N | Y | P | 2.9, -1.4 | PKA, PKG | Protein Kinase A | [PKA](http://kinasephos.mbc.nctu.edu.tw/help.html#kinase) | >1 | E |
|  | 1287 | C | N | N | - | - | - | - | - | - | E |
|  | 1302 | CS | N | N | - | - | CKI | - | - | - | E |
|  | 1319 | C | N | P | - | - | - | - | - | - | B |
|  | 1334 | C | N | Y | - | - | - | - | - | - | E |
| Tyrosine | 36 | NC | N | Y | - | - | EGFR | - | - | - | B |
|  | 75 | NC | Y | P | - | - | - | - | - | - | B |
|  | 76 | NC | N | Y | - | - | - | - | - | - | B |
|  | 111 | NC | N | Y | - | - | - | - | - | - | B |
|  | 116 | NC | N | N | - | - | - | - | - |  | B |
|  | 121 | NC | N | Y | P | 0.2 | SRC, INSR | PDGFR Kin | [Jak](http://kinasephos.mbc.nctu.edu.tw/help.html#kinase) | <1 | B |
|  | 136 | NC | N | P | - | - | SRC | - | - | - | B |
|  | 184 | NC | Y | Y | - | - | SRC, INSR | - | - | - | B |
|  | 194 | NC | N | N | - | - | - | - | - | - | B |
|  | 217 | NC | N | N | - | - | - | - | - | - | B |
|  | 459 | NC | N | P | P | - | - | Abl SH2 | - | >1 | E |
|  | 503 | NC | N | Y | - | - | SRC | - | - | - | B |
|  | 540 | NC | N | P | P | - | - | PDGFR Kin, Lck SH2, p85 SH2 | - | <1 | E |
|  | 542 | NC | N | Y | P | 3.3, -2.3 | - | Insulin Receptor Kinase, p85 SH2 | [INSR](http://kinasephos.mbc.nctu.edu.tw/help.html#kinase), [Syk](http://kinasephos.mbc.nctu.edu.tw/help.html#kinase) | <1 | E |
|  | 556 | NC | N | P | - | - | - | - | - | - | E |
|  | 576 | NC | N | P | - | - | - | - | - | - | E |
|  | 598 | NC | N | Y | - | 0.2 | INSR | - | [INSR](http://kinasephos.mbc.nctu.edu.tw/help.html#kinase) | - | E |
|  | 625 | NC | N | P | - | - | - | - | - | - | E |
|  | 628 | NC | N | N | - | - | - | - | - | - | E |
|  | 632 | NC | N | Y | P | 0.6, -0.8 | INSR | PDGFR Kin, Fgr Kinase, Src Kinase | [INSR](http://kinasephos.mbc.nctu.edu.tw/help.html#kinase), [Syk](http://kinasephos.mbc.nctu.edu.tw/help.html#kinase) | >1 | E |
|  | 653 | NC | Y | Y | P | 2.6, 2.4, 5.0 | EGFR, INSR | PDGFR Kin, p85 SH2 | [INSR](http://kinasephos.mbc.nctu.edu.tw/help.html#kinase), [Syk](http://kinasephos.mbc.nctu.edu.tw/help.html#kinase), [Other_MDD](http://kinasephos.mbc.nctu.edu.tw/help.html#kinase) | <1 | E |
|  | 675 | NC | Y | Y | P | -1.2, 5.8, 1.4 | INSR | PDGFR Kin, Insulin Receptor Kinase, p85 SH2 | [Src](http://kinasephos.mbc.nctu.edu.tw/help.html#kinase), [INSR](http://kinasephos.mbc.nctu.edu.tw/help.html#kinase), [Syk](http://kinasephos.mbc.nctu.edu.tw/help.html#kinase) | >1 | E |
|  | 727 | NC | N | Y | - | -4.5 | - | - | [INSR](http://kinasephos.mbc.nctu.edu.tw/help.html#kinase) | - | E |
|  | 742 | NC | Y | Y | P | 2.1, -3.2 | EGFR, INSR | p85 SH2 | [INSR](http://kinasephos.mbc.nctu.edu.tw/help.html#kinase), [Syk](http://kinasephos.mbc.nctu.edu.tw/help.html#kinase) | <1 | E |
|  | 766 | NC | N | Y | P | -3.0, 5.4 | EGFR | PDGFR Kin, Fgr Kinase, PLCg N-terminal SH2, Grb2 SH2 | [Syk](http://kinasephos.mbc.nctu.edu.tw/help.html#kinase), [Other_MDD](http://kinasephos.mbc.nctu.edu.tw/help.html#kinase) | <1 | E |
|  | 803 | NC | N | P | - | - | - | - | - | - | E |
|  | 810 | NC | N | N | - | - | - | - | - | - | E |
|  | 814 | NC | N | Y | - | - | INSR | - | - | - | E |
|  | 823 | NC | Y | Y | P | 1.3, -3.4 | INSR | PDGFR Kin, p85 SH2 | [INSR](http://kinasephos.mbc.nctu.edu.tw/help.html#kinase), [Syk](http://kinasephos.mbc.nctu.edu.tw/help.html#kinase) | <1 | E |
|  | 907 | C | N | Y | P | - | - | PLCg C-terminal SH2 | - | >1 | E |
|  | 919 | C | Y | Y | P | 3.9, 5.6 | - | PDGFR Kin, Fgr Kinase, PLCg N-terminal SH2, Grb2 SH2 | [INSR](http://kinasephos.mbc.nctu.edu.tw/help.html#kinase), [Other_MDD](http://kinasephos.mbc.nctu.edu.tw/help.html#kinase) | <1 | E |
|  | 978 | C | N | Y | P | 1.2 | - | PDGFR Kin, Grb2 SH2, PLCg N-terminal SH2 | [INSR](http://kinasephos.mbc.nctu.edu.tw/help.html#kinase) | >1 | E |
|  | 1014 | C | N | P | P | - | - | PDGFR Kin | - | >1 | E |
|  | 1042 | C | N | P | P | - | - | Abl Kinase | - | >1 | E |
|  | 1072 | C | N | Y | P | 3.6, -3.0 | EGFR, INSR | PDGFR Kin, Lck SH2, Fyn SH2, p85 SH2 | [INSR](http://kinasephos.mbc.nctu.edu.tw/help.html#kinase), [Syk](http://kinasephos.mbc.nctu.edu.tw/help.html#kinase) | >1 | E |
|  | 1253 | C | N | P | - | -0.5 | - | - | [INSR](http://kinasephos.mbc.nctu.edu.tw/help.html#kinase) | - | E |
|  | 1320 | C | N | Y | - | -4.4 | - | - | [INSR](http://kinasephos.mbc.nctu.edu.tw/help.html#kinase) | - | B |
